# Quantifying the uniqueness and divisiveness of presidential discourse

**DOI:** 10.1093/pnasnexus/pgae431

**Published:** 2024-10-07

**Authors:** Karen Zhou, Alexander A Meitus, Milo Chase, Grace Wang, Anne Mykland, William Howell, Chenhao Tan

**Affiliations:** Department of Computer Science, The University of Chicago, 5730 S Ellis Ave, Chicago, IL 60637, USA; Department of Computer Science, The University of Chicago, 5730 S Ellis Ave, Chicago, IL 60637, USA; Harris School of Public Policy, The University of Chicago, 5730 S Ellis Ave, Chicago, IL 60637, USA; Department of Computer Science, The University of Chicago, 5730 S Ellis Ave, Chicago, IL 60637, USA; Department of Computer Science, The University of Chicago, 5730 S Ellis Ave, Chicago, IL 60637, USA; Harvard University, Massachusetts Hall, Cambridge, MA 02318, USA; Harris School of Public Policy, The University of Chicago, 5730 S Ellis Ave, Chicago, IL 60637, USA; Department of Political Science, The University of Chicago, 5730 S Ellis Ave, Chicago, IL 60637, USA; Department of Computer Science, The University of Chicago, 5730 S Ellis Ave, Chicago, IL 60637, USA; Harris School of Public Policy, The University of Chicago, 5730 S Ellis Ave, Chicago, IL 60637, USA

**Keywords:** presidential speech, uniqueness metrics, large language models, divisive word lexicon, Donald Trump

## Abstract

Do American presidents speak discernibly different from each other? If so, in what ways? And are these differences confined to any single medium of communication? To investigate these questions, this paper introduces a novel metric of uniqueness based on large language models, develops a new lexicon for divisive speech, and presents a framework for assessing the distinctive ways in which presidents speak about their political opponents. Applying these tools to a variety of corpora of presidential speeches, we find considerable evidence that Donald Trump’s speech patterns diverge from those of all major party nominees for the presidency in recent history. Trump is significantly more distinctive than his fellow Republicans, whose uniqueness values appear closer to those of the Democrats. Contributing to these differences is Trump’s employment of divisive and antagonistic language, particularly when targeting his political opponents. These differences hold across a variety of measurement strategies, arise on both the campaign trail and in official presidential addresses, and do not appear to be an artifact of secular changes in presidential communications.

**Significance Statement**

While presidential discourse attracts considerable attention from popular media and scholars alike, efforts to computationally compare such rhetoric have been limited to lexical methods. This paper proposes a novel suite of metrics to advance such analyses and identify new findings. In particular, we leverage large language models to establish an original metric of uniqueness, develop a new lexicon for divisive speech, and introduce a comparative framework for the portrayal of political opponents. We then apply these methods to a rich assembly of campaign speeches, presidential debates, and official States of the Union addresses. Across all these datasets, we find that Donald Trump’s political rhetoric is unique among modern presidents, and is defined, in part, by his use of antagonistic language, particularly when directed at political opponents.

## Introduction

The rise of the modern presidency is defined, in no small measure, by the chief executive’s changing relationship to the American public. In what Jeffrey Tulis (1) calls the “rhetorical presidency,” modern presidents are expected to routinely stand before the public in order to explain, persuade, inform, and instruct. How precisely they do so, though, is open to interpretation. While certain norms of communication govern their behavior, presidents have a fair measure of discretion to speak as they choose.

When fulfilling their oratory duties, do modern presidents adhere to a common script? Or do some presidents defy rhetorical conventions and speak in ways that, at least among themselves, are novel and surprising? Leveraging recent advances in large language models (LLMs) (2), we develop a new quantifier of uniqueness by directly measuring the unpredictability of language patterns. In addition, we incorporate more standard lexical techniques to examine a prevalent yet understudied construct, divisiveness (3). We operationalize *divisiveness* as language that is intended to impugn and delegitimize the speaker’s target, and we develop a new lexicon for such speech. Furthermore, we introduce a comparative framework for assessing mentions of opponents, which are especially prevalent in presidential debates.

We use our proposed tools to analyze large and diverse corpora of presidential speech. In doing so, we are able to both characterize the overall distinctiveness of presidents’ speech patterns and examine specific qualities that previous research has overlooked. Moreover, we can distinguish general speech patterns from those directed towards one’s political opponents.

In nearly all of our analyses, Donald Trump appears as a clear outlier. On the campaign trail, in presidential debates, and in official presidential addresses, we find, Trump’s speech patterns routinely differ from those of all recent presidents —lending credence to Kurt Anderson’s observation that, “The version of English [Trump] speaks amounts to its own patois, with a special vocabulary and syntax and psychological substrate” (4).

Like previous scholars (5–7), we find that Trump tends to communicate in shorter, more simplistic sentences. But whereas previous quantitative research on presidential rhetoric relied exclusively on lexicons (e.g. (6)) and sentiment classification (e.g. (8)), which necessarily disregard contextual information included in the body of speeches, our methods are able to show that Trump speaks in ways that are holistically different from all modern presidents. These differences are pervasive and large—so much so, in fact, that the observed differences between Trump and his fellow Republicans exceed those between Republicans and Democratic presidents. Contributing to these differences, we show, is Trump’s tendency to speak in ways that are especially divisive, particularly when focusing on his political opponents.^a^ These findings, moreover, are robust to a variety of measurement strategies, arise across rich and diverse corpora of texts, and do not appear to be an artifact of secular time trends.

## Methodology

### Data

Our research investigates three genres of political speech: presidential debates in general elections since 1960, State of the Union (SOTU) speeches since 1961, and a sample of campaign speeches assembled by the American Presidency Project (13). Debates and SOTU speeches are generally standard across presidents and time, so all available documents are included in our main analyses.^b^ Publicly available campaign documents, however, are imbalanced and not comprehensive over the same time frame, so these corpora are limited to speeches delivered within one month of Election Day in every presidential election since 2008. Table 1 provides summary statistics of the final datasets for which we present results.^c^

**Table 1.** Overall statistics of the three data types we use to present our main findings.

	DEBATES	SOTU	CAMPAIGN
# speeches	35	67	187
# sentences	35,096	22,775	36,295
date range	1960–2020	1961–2022	2008–2020
# candidates compared	19	11	6
party ratio (Dem:Rep)	10:9	5:6	3:3
avg. sents/candidate	1,449	2,070	5,701
avg. candidate sent len.	18.76	24.42	17.62
avg. speeches/candidate	3.6	6.1	31.2

**Fig. 1.**
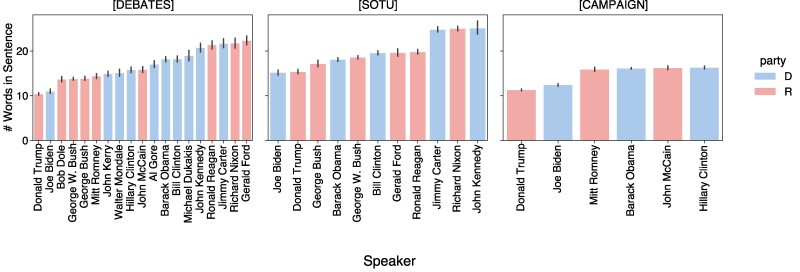
Distribution of sentence lengths among all speakers, across debates, SOTU, and campaign data (error bars represent 95% CI). Donald Trump tends to use the shortest sentences on average.

Figure 1 shows the distribution of sentence lengths from each speaker across the datasets. Further details of how we collected these datasets can be found in Materials and Methods. From the outset, however, we note that Trump tends to speak in markedly shorter sentences than do other presidents. Whereas Trump’s sentences range from 10.4 to 14.5 words in the three data sources, the overall averages range from 17.6 to 24.4 words. And when comparing presidents within each data source, Trump registered the single lowest number of average words per sentence among all presidents within debates and campaigns, and the second lowest number within SOTU addresses.

### Quantifying the uniqueness of political speech

We quantify the uniqueness of political speeches using three complementary approaches: (i) a novel metric based on LLMs; (ii) a new resource of lexicons for divisive speech; and (iii) a comparative framework for the portrayal of political opponents involving lexical features. Together, these three approaches allow for a robust and multifaceted comparison of speech patterns that involve global assessments beyond lexical comparisons and specific evaluations of divisiveness. We briefly introduce the intuition behind each approach below.

####  

##### LLM-based uniqueness

Large language models, like the GPT family of models, have received widespread attention for their abilities to statistically characterize the complex structures of natural language text. LLMs can measure the predictability of text by calculating the likelihood of the next word or sequence of words in a given context, to produce a measure known as “perplexity,” which is typically used to evaluate the quality of LLMs (14, 15). To control for the length of a text, standard perplexity measures can be supplemented with measures of “bits-per-character” (BPC) (16).

Contributing to this literature, we propose a metric of “uniqueness” based on the ability of LLMs to estimate the probabilities of word sequences, and we then use these estimates to compare political speech from various presidents and presidential candidates, whether delivered from the White House or on the campaign trail. Specifically, from a pool of presidential candidates, we determine how likely the speech of one speaker is to be produced by the others. A positive value for one candidate’s sentence uniqueness, therefore, suggests that other candidates are unlikely to say it. The larger this value, the less likely other candidates are to do so.

The advantage of this metric is its consideration of the preceding context of given speech. Rather than examine words or phrases in isolation, this metric considers the order in which they appear, and thereby provides a much more nuanced characterization of speech patterns. The precise meaning of the scores, however, may be difficult to interpret because they do not reveal the exact features that make a speech distinctive, and because they only allow for comparisons within a specified pool of speakers. Technical details on the construction of this metric are included in the Materials and Methods.

##### Divisive speech lexicon

To analyze the actual content of language used by presidents and presidential candidates, a “divisiveness” lexicon is created and applied to each dataset. We define language as “divisive” if it intends to impugn and delegitimize the speaker’s target, e.g. by attacking their intelligence, integrity, or intentions. Examples of divisive accusations include “racist,” “dishonest,” “corrupt,” or “ridiculous.” Such labels are expressly designed to put the target on defense and accentuate differences and distance between parties.

Our definition of divisive is distinct from other commonly analyzed constructs such as political “polarization,” which encompasses language that is associated more with one side than the other but is otherwise agnostic about its valence (17). Meanwhile, speech may be divisive without being “toxic,” which contains hateful, abusive, or offensive content (18). Personal attacks can also be categorized as a form of toxic language, which prior work has examined primarily in the context of online, written communication (19). Nor is our measure of divisiveness the simple antonym of traditional notions of politeness (20), which usually involve honoring social conventions, showing gratitude, paying compliments, avoiding complaints and curses, and respecting the listener’s autonomy with the use of softening statements and hedges. More than just impolite or insulting, divisive speech, as we conceive it, is explicitly intended to serve the political purposes of delegitimization, marginalization, and distancing between speaker and target.

To the best of our knowledge, ours is the first divisive speech lexicon. As we explain in further detail in the Material and Methods section, this lexicon consists of 178 words that four researchers independently reviewed to be qualitatively “divisive” in political speech. A strength of this lexicon-based analysis is its easy interpretability and applicability. Divisive words may be used by candidates from any political party in a wide variety of settings, are readily identified, and, as we subsequently show, are broadly agreed upon by coders. As with all lexical approaches, however, the measure is inherently limited by the subjective nature of lexical evaluations and the lack of contextual consideration. Our resource, like all lexicons, by itself does not account for surrounding context like negation or valence. Strategies like pairing our lexicon with part-of-speech taggers may be leveraged for downstream tasks.

##### References to political opponents

Our analysis further expands upon prior work by culling the subset of sentences that explicitly refer to political opponents. We specifically examine presidential debates, in which we define “opponents” here to be either the debate partner or their party. The methodology of tagging opponent mentions is described in the Materials and Methods section.

Once speech referring to opponents is distinguished, we employ the Fightin’ Words (FWs) method (21) to identify words more strongly associated with opponent mentions; we extend this comparison across multiple candidates by calculating the overlap of each entity’s corresponding word sets with those of all presidents and presidential candidates. Intuitively, a greater overlap indicates that other candidates use similar rhetoric in opponent mentions, while a smaller overlap indicates that other candidates do not use similar rhetoric in opponent mentions. This metric thus provides a novel measure for quantifying a candidate’s distinctiveness with respect to their portrayal of opponents by combining lexical and graph analysis. The results are readily interpretable and clarify the distinctive qualities of language used to characterize one’s opponent. The metric is similarly constrained by the pool of candidates and the finite selection of descriptors.

## Results

### Donald Trump is unique among all presidential candidates in all types of speech

####  

##### LLM-based uniqueness

**Fig. 2.**
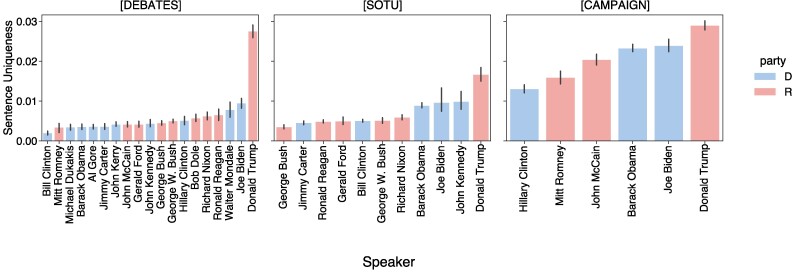
Average sentence uniqueness for each speaker, across all data types. Higher bars indicate greater uniqueness, i.e. that speaker’s speech is less likely to be uttered by other candidates. Trump is the most distinctive speaker among these candidates for debates and SOTU speeches. Error bars correspond to the 95% CI.

**Fig. 3.**
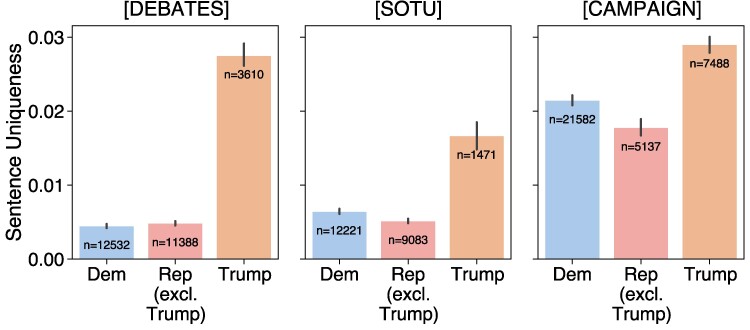
Trump’s average sentence uniqueness scores compared to the others aggregated by party. The number of sentences is denoted on each bar (with error bars representing 95% CI). Trump is significantly distinct from other Republicans. The uniqueness scores of the remaining Republicans are closer to those of Democrats than with Trump. For SOTU and campaign speeches, Republicans (without Trump) are less unique than Democrats on average.

We start by presenting results based on the LLM-based uniqueness metric. We find that Trump is the most distinctive speaker in debates and SOTU speeches (see Figs. 2 and 3). Additionally, among campaign speeches analyzed from 2008 onwards, Trump speaks in ways that stand apart from all other candidates. By comparing candidate speech patterns aggregated by party, Fig. 3 shows that Trump is more distinctive than his fellow Republicans for all types of speech. Indeed, the observed difference between Democratic and Republican candidates is minor compared to the gap observed between Trump and everyone else.

**Fig. 4.**
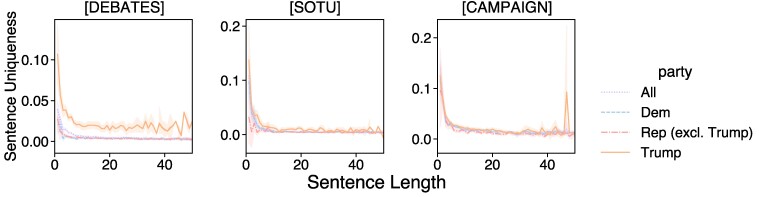
Sentence uniqueness across different sentence lengths, for Trump, all other Republicans, and Democrats. For debates and SOTU, Trump is consistently more distinctive across all sentence lengths.

While Trump’s speech is characterized by shorter sentences (Fig. 1), we confirm that Trump’s uniqueness is consistent across sentences of all lengths in Fig. 4. We also note that Biden has similarly short sentences on average as Trump, but his uniqueness scores are close in magnitude to those of the other candidates, particularly in debates and SOTU addresses.

Furthermore, we find that there is minimal correlation between our uniqueness metric and standard simplicity scores, suggesting that the language model is not conflating uniqueness with language complexity (see Supplementary material).

##### Further extensions and robustness checks

When aggregating over presidential terms and years, Trump appears as slightly more distinctive in 2016 than in 2020 for debates, while his SOTU and campaign speeches increase in uniqueness over the years. Overall, he remains consistently more distinctive in both election cycles than the other candidates under consideration (see Supplementary material). Prior to Trump’s first election cycle, there are no clear temporal trends to suggest that uniqueness has been increasing over time. Moreover, when comparing the uniqueness scores of the top decile of unique sentences for each speaker, we again find that Trump is the most distinctive speaker in all our samples of political speech (see Supplementary material).

##### Score validation

The results presented in the main paper are based on the OpenAI GPT-2 model, which was the state-of-the-art open-source causal language model at the start of this project (22). We are interested in causal language modeling, i.e. the predictability of text in a *forward sequential manner*. Causal language models are more appropriate for our task of scoring uniqueness, as opposed to masked language models (e.g. BERT, RoBERTa) which are designed to predict missing tokens within a sequence based on *bidirectional* context (and thus excel at text classification). We validate our results with more recent and powerful LLMs like Gemma 2B (23) and Phi1-5b (24) as well in the Supplementary material. The results from these recent LLMs reaffirm our original findings; that is, Trump is consistently identified as the most unique speaker among modern presidential candidates in all types of speech.

### Trump speaks most divisively

####  

##### Divisive word lexicon

**Fig. 5.**
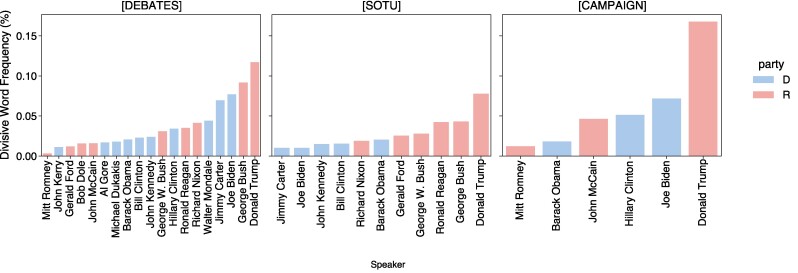
Overall percentage of words used that are divisive. Trump uses the most words from our divisive lexicon, in all types of speech.

Leveraging our divisive word lexicon, we measure its usage across the different types of presidential speech. The frequency of lexicon word appearances is calculated for each candidate across each dataset and is shown in Fig. 5. Word frequencies are calculated as the number of times a divisive word from the lexicon is spoken by a speaker in a given dataset (debates, SOTU, campaign speeches) divided by the total number of words spoken by the speaker in that in dataset. Both the lexicon words and the speech data are processed to remove contractions and punctuation. Frequency trends provide a macroscopic look at the usage of divisive language over time. In the Supplementary material, temporal plots and heatmaps of divisive word usage provide a more granular look and do not show strong trends over time.

One common trend shown by the frequency plots for each dataset is Donald Trump’s relatively high usage of words from the divisiveness lexicon compared to other speakers. In all three datasets, Donald Trump’s speeches rank highest in divisive word usage compared to the other candidates analyzed. Examples of Trump’s most frequently used words from this lexicon usage are shown in Table 2. He is most verbosely divisive in debates and campaign speeches, uttering terms like “crazy,” “corrupt,” and “stupid” with high frequency. The contexts of debates and campaigns are more combative than SOTU addresses, which is reflected in the higher levels of divisive language in those two mediums. Table 3 contains a selection of Trump‘s divisiveness in context; additional example sentences from various speakers can be found in the Supplementary material.

**Table 2.** Top 10 divisive words used by Trump in each type of speech.

DEBATES	SOTU	CAMPAIGN
stupid (14)	cruel (3)	crazy (135)
racist (14)	vile (3)	corrupt (111)
disgrace (12)	ruthless (2)	stupid (69)
corrupt (8)	foolish (2)	dishonest (53)
disgraceful (8)	corrupt (2)	disgrace (45)
ridiculous (6)	reckless (2)	ridiculous (31)
ashamed (6)	savages (1)	racist (27)
stupid (6)	ugly (1)	incompetent (25)
filthy (6)	outrageous (1)	stupidity (22)
dishonest (6)	ridiculous (1)	ashamed (22)

Raw counts of usage are denoted in parentheses next to each word.

Across all debates and campaign speeches, which are the two corpora with greatest prevalence of divisive language, sentences that use divisive words are more unique (see Supplementary material). The Spearman correlation coefficient between uniqueness and divisive word usage is 0.01 in debates and campaigns (p<0.05 in campaigns and p=0.30 in debates). The weak, positive correlation suggests that divisiveness amounts to only a small part of the overall uniqueness of any speech pattern.

**Table 3.** Example sentences from debates and campaigns, with preceding context, spoken by Trump that use words from the divisive lexicon. Bolded words are matched with our divisiveness lexicon.

**DEBATES**:
*Trump:* Let me tell you something.
*Trump:* You take a look at Mosul.
*Trump:* The biggest problem I have with the **stupidity** of our foreign policy, we have Mosul.
—
*Trump:* I was at a little Haiti the other day in Florida.
*Trump:* And I want to tell you, they hate the Clintons, because what’s happened in Haiti with the Clinton Foundation is a **disgrace**.

**CAMPAIGN**:
*Trump:* We’re going to bring back the miners and the factory workers and the steel workers.
*Trump:* We’re going to put them back to work.
*Trump:* The economic policies of Bill and Hillary Clinton have destroyed manufacturing in your state and throughout the entire country.
*Trump:* The **corrupt** Clintons gave us NAFTA.
—
*Trump:* The fact is, this is the single most important election in the history of our country.
*Trump:* And sleepy Joe Biden’s made a **corrupt** bargain.
*Trump:* You saw the bargain he made, in exchange for his party’s nomination, which he shouldn’t have gotten because if Pocahontas got out one day early, I’d be running against **Crazy** Bernie, which would have been okay, too.

### References to political opponents in speech

In addition to examining overall speech patterns, we can also extract the portions that reference political opponents. Next, we show that Trump is more likely to mention his political opponents than are other presidential candidates and that when doing so, Trump uses particularly distinctive language.

####  

##### Rates of opponent mentions

As one might expect, candidates routinely mention their opponents in debates and only rarely in SOTU addresses. Overall, rates of sentences that mention opponents for debates, SOTU, and campaign speeches are 20.60%, 0.83%, and 6.95%, respectively (see Supplementary material). Trump has the highest rate of opponent mentions in debates.

**Fig. 6.**
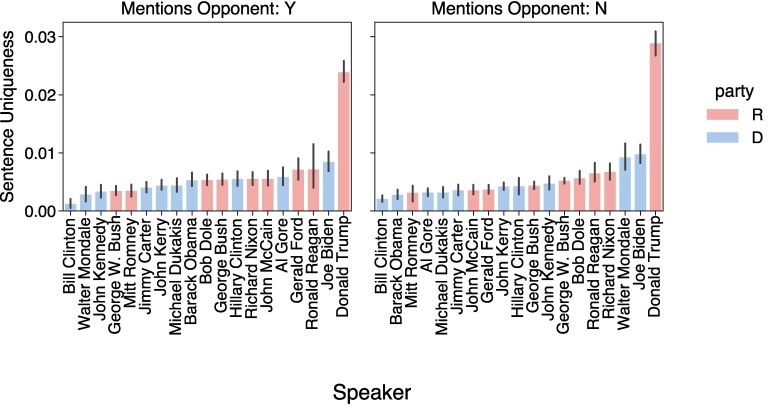
Uniqueness of speech broken down by opponent mentions, for debates (the error bars represent 95% CI). Trump is most distinctive regardless of whether he references an opponent or not.

Since the opponent mentions are more common in presidential debates, we revisit our LLM-based measure for the debates to confirm that Trump is not unique simply because he is more likely to mention opponents. Fig. 6 shows that the LLM-based uniqueness score rankings are relatively consistent across opponent and nonopponent mentions. Moreover, Trump’s language is consistently unique in these debates, whether or not he calls out an opponent. Concurrently, sentences that refer to an opponent tend to be more distinct than those that do not (see Supplementary material). And in debates, we additionally find that sentences that mention opponents contain significantly higher frequencies of divisive words (see Supplementary material). If you are looking to isolate the distinguishing characteristics of a candidate’s speech patterns, therefore, you would do well to focus on how they talk about their political opponents.

##### FWs adjective overlap

To identify which *adjectives* are most strongly associated with describing opponents for each candidate, we calculate the odds ratios between opponent mention sentences and nonopponent mention sentences, i.e. FW (21). After these odds ratios are calculated, we compare the words most commonly associated with opponent mentions across candidates and calculate an overlap score. This *FW overlap metric* for each candidate is calculated as the average number of speakers who share each of the candidate’s top-*n* FW adjectives. See the Materials and Methods section for further details on the construction of this metric.

**Fig. 7.**
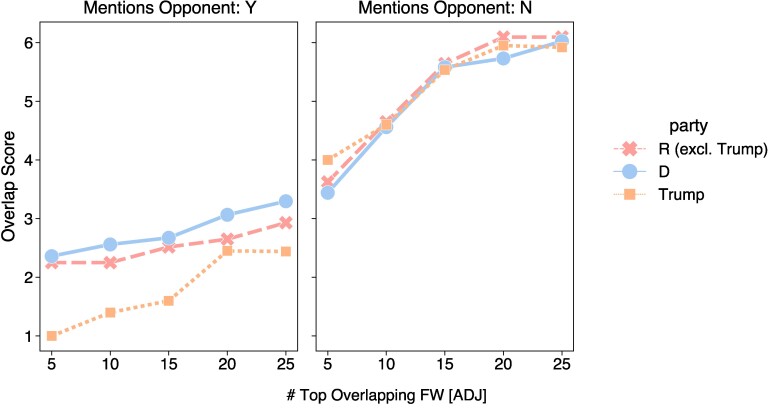
FW overlap scores across different top-*n* thresholds, comparing Trump and the other candidates aggregated by party.

**Fig. 8.**
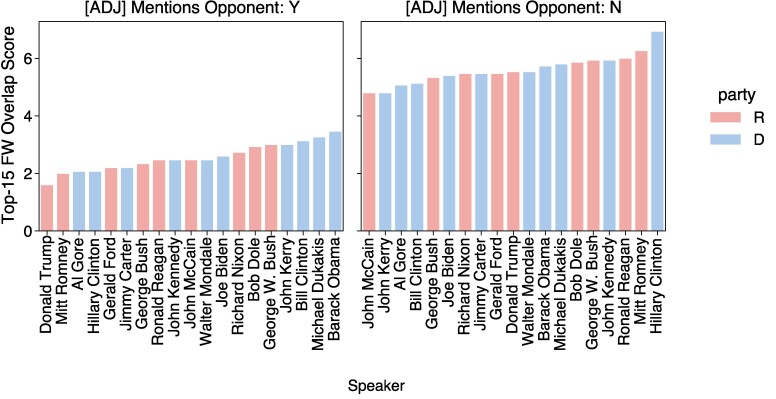
Top-15 FW overlap score in debates for each candidate. Trump’s FW associated with opponent mentions generally have the lowest overlap in adjective usage compared to other candidates, which is another indication that his language is distinctive.

Intuitively, the lower the FW overlap score for a candidate, the fewer adjectives that candidate uses in common with others when describing their political opponents. For example, a low FW overlap metric indicates that the words a politician uses most frequently to describe an opponent are distinct from those of other candidates. From Fig. 7, we see that Trump has the lowest FW overlap in debates on average across different top-*n* thresholds. Among adjectives that Trump uses in references to opponents, his top-*n* FW are consistently distinct for n=5 to n=25.^d^ Fig. 8 ranks the speakers by their FW overlap score at the top-15 threshold and shows that Trump has the lowest FW overlap scores when mentioning opponents (additional top-*n* plots can be found in the Supplementary material). Trump has a higher overlap of FW descriptors when not referencing opponents, indicating that he is more distinctive for how he *describes* his opponents.

Examples of Trump’s top-25 FW adjectives can be seen in Table 4; the left panel includes language used to describe his political opposition, while the right panel includes references to his own party and allies. In addition to attacking opponents (e.g. “disgraceful”), Trump tends to use fairly simplistic adjectives like “massive” and “super.”^e^

**Table 4.** Top-25 FW adjectives said by Donald Trump in debates, either in reference to opponents or not.

Mentions opponents: Y	Mentions opponents: N
left (2.07), long (1.85), tough (1.80), own (1.79), ok (1.79), bigger (1.79), worse (1.66), radical (1.57), super (1.50), real (1.50), effective (1.47), xenophobic (1.46), liberal (1.46), disgraceful (1.46), massive (1.33), single (1.33), political (1.09), last (1.09), economic (1.08), short (1.08), huge (1.04), various (1.04), red (1.04), upset (1.04), back (1.04)	good (−3.63), great (−3.25), important (−2.36), more (−2.08), inner (−2.00), strong (−1.99), right (−1.84), proud (−1.80), other (−1.79), expensive (−1.64), old (−1.53), big (−1.53), better (−1.50), greatest (−1.44), young (−1.41), sad (−1.41), able (−1.31), much (−1.31), African (−1.27), fine (−1.27), beautiful (−1.27), tougher (−1.18), nice (−1.18), least (−1.12), sure (−1.12)

Z-scores are provided in parentheses.

## Discussion

In this work, we proposed novel approaches to quantify the uniqueness and divisiveness of presidential discourse. Our results show that whether on the campaign trail, the debate stage, or the official dais of the House of Representatives, Trump speaks differently from all modern presidents and presidential candidates. We confirm his distinctiveness for speaking in shorter, simpler, and more repetitive sentences. We further quantify the uniqueness of his overall speech compared to that of other presidential candidates. Finally, we demonstrate that Trump uses language that is more divisive, antagonistic, and explicitly focused on his political opponents.

The differences between Trump and other candidates do not appear to be an artifact of secular communication trends (see Supplementary material), whether in the general coarsening of public discourse or the tendency to speak in simpler sentences. Across multiple points of comparison, observed differences between Trump and other contemporary candidates appear every bit as large as those between Trump and candidates from the 1960s and 1970s.

Our findings, of course, come with a variety of limitations. The campaign data, for instance, include samples from only the most recent candidates and exclude certain communication formats, such as interviews. None of our datasets cover instances when surrogates (family members, vice presidents, etc.) speak on behalf of a candidate. While other works have examined Trump’s tweets (25), we do not assess any of his social media postings for lack of comparable data for other candidates, especially older ones. Furthermore, our data collection ends at 2022, excluding more recent remarks from Trump that have been flagged in the media for their escalated divisiveness (26).

Our research invites a variety of extensions. Given that values of the LLM-based uniqueness metric can only be understood relative to the elements of a selected sample, future work would do well to compare Trump to other speakers, such as populist leaders in other countries. Just as we investigate divisiveness as a new dimension of political speech with particular resonance for Trump’s rise to power, other scholars might evaluate other dimensions of political speech, such as its analytical sophistication or propensity to endorse racial, gender, or sexual stereotypes. Additionally, future research might explore the possibility of contagion effects from Trump’s speech patterns to other political figures, as politicians periodically emulate and influence one another.

We also recognize that all of our metrics come with tradeoffs, which we have elucidated throughout this essay. Still, the tools we develop can be applied to a wide variety of settings. While we use them to compare speech patterns among presidents and presidential candidates, they can just as easily be deployed to analyze the language used by any public officials. And in addition to characterizing overall differences in speech patterns, we illuminate ways of assessing particular qualities that appear in either stand-alone speeches or interactive exchanges.

The substantive findings presented herein, moreover, establish the uniqueness of Trump’s speech patterns, just as they reveal particular qualities that distinguish Trump from all modern US presidential candidates. More research, of course, is required to map these speech patterns into larger political strategy. Nonetheless, we conjecture that these qualities broadly contribute to Trump’s enduring appeal as a populist who unabashedly denounces established political enemies in a historical period of acute polarization, distrust, and division.

## Materials and methods

### Data collection

Each dataset is scraped from the American Presidents Project (APP) database (13). In general, all text for each speech is scraped and tagged with its speaker. In addition, some metadata are collected, like the date and title of the speech or document. In order to properly annotate speakers, particularly in the debate speeches, the speaker’s name is identified based on a variety of factors like special text styling, string matching, and html formatting. In addition, audience annotations, like “laughter” and “applause,” are filtered out from the data to the best of our ability. After scraping, the data are subsampled and reviewed to ensure quality and correct speaker annotations.

####  

##### Debates data

The Presidential debates data include General Election debates from 1960 to the present day and Primary Election debates since 2000. For the purposes of this analysis, we exclude primary debates and any general election debates that featured vice-presidential candidates. Sentences spoken by candidates who were not the nominee for either the Republican or Democratic party are also excluded (e.g. Ross Perot in 1992, moderators, and audience members).

##### SOTU data

Because we focus on modern presidents, State of the Union addresses from 1961 onwards are used in deriving our main results. The SOTU dataset includes a few speeches that are not officially considered State of the Union addresses but functionally operate as such. Beginning with Reagan, recent presidents have started addressing a joint session of Congress shortly after their inaugurations. According to the American Presidency Project, “it is probably harmless to categorize these as State of the Union messages (as we do). The impact of such a speech on public, media, and congressional perceptions of presidential leadership and power should be the same as if the address was an official State of the Union”.^f^

##### Campaigns data

All campaign documents from 1932 to 2020 are collected initially. We manually identify keywords that indicate campaign speeches of interest and filter speech titles based on those keywords. Keywords selected are: “remarks,” “speech,” and “address.” Manually removed documents include: speeches with reporters, question/answer format speeches, press releases, town halls, press releases, co-appearances with other politicians and spouses, etc.

Subsequently, we apply clique filtering to ensure that any duplicate speeches are removed. It is common that a candidate has a stump speech that is delivered at multiple events, and to diversify and balance the dataset, we remove such duplicates. These de-duplicated data are used for model training.

After filtering, our final campaign dataset includes only speeches delivered within the month before election day and only from candidates since 2008. This smaller subset is used to generate the results presented in the main paper.

### Sentence and opponent mention tagging

After each dataset is filtered, sentences are tagged as mentioning an opponent or not through an automated process. Sentences are classified as definitely including an opponent mention, possibly including an opponent mention, or not including an opponent mention based on keywords and the presence of parts of speech. For debates, “opponents” are identified by the name(s) of the debate partner or their party. After automatically tagging the debates dataset, the sentences that are labeled as possibly including an opponent mention are manually reviewed by an expert team of four researchers. The team compares a subset of pairwise overlapping ratings for consistency. The Cohen’s *κ* for inter-coder agreement is roughly 0.8, which indicates substantial agreement.

For SOTU, opponent mentions only include those of other presidential candidates; names of candidates who never held presidential office are not categorized as mentions of opponents, in contrast to the debates dataset. Following a manual review of selected speeches from the SOTU dataset, it is concluded that references to nonpresidential figures are much more neutral and considerably less frequent in State of the Union speeches than they are in debate contexts.

For campaign data, we automatically tag opponents using the names of the final party candidates from the opposing party of the speaker. Opposing-party candidates from the primaries are not automatically tagged.

The strict guidelines for automatic tagging ensure high precision of the labels. Limitations of this automatic tagging method include lack of coreference resolution and difficulty of measuring recall.

### Language model training and analysis

We fine-tune a pretrained, 124M parameter GPT-2 model using the huggingface (22) and PyTorch Lightning (27) libraries for each of the three data types, resulting in three different language models:

- Lm_Debates_, trained on 35 debates (35,096 sentences) from 1960 to 2020
- Lm_Sotu_, trained on 246 speeches (69,630 sentences) from 1790 to 2022
- Lm_Campaign_, trained on 640 documents (83,038 sentences) from 1932 to 2020

To preprocess data for training, we parsed each speech into sentences, prefixed each sentence with the speaker prompt (e.g. “Donald Trump:,” and masked any named entities (as identified by spaCy NER tagger) with a <ENT> mask.^g^ We fine-tune each model for 10 epochs on all available corresponding data with a learning rate of 5e-5. Validation with larger, more recent LLMs is shared in the Supplementary material.

### BPC/predictability and uniqueness scores

Consider a set of presidents or candidates $C=\{c_{1},\ldots ,c_{n}\}$ who each have a corresponding set of sentences $S=\{S_{1},\ldots ,S_{n}\}$ where each set $S_{i}=[s_{i}^{1},\ldots ,s_{i}^{m}]$, for each of the three data types.

We use BPC, also known as bits-per-byte, as a proxy for “predictability” of a sentence. Lower BPC values correspond to higher predictability. We use BPC instead of perplexity or loss directly to account for variation in tokenization techniques.

We calculate BPC as follows. From our fine-tuned models, we are able to obtain *loss* values, $L(t_{i})$ for each token $t_{i}$ in an input. For some sentence of tokens $s=(t_{1},t_{2},\ldots ,t_{k})$ where len(s) denotes the number of characters in *s*:


$$BPC(s)=\frac{1}{\text{len}(s)}\sum_{i=1}^{k}L(t_{i})$$

In other words, BPC(s) is the sum of cross-entropy losses of each token in a sentence *s*, divided by the number of characters (bytes) in the *s*. We calculate the BPC(s) of a sentence *s* with a context window size of 512 tokens. That is, preceding sentences of the one in question are provided to obtain the most representative score of its predictability.

We first obtain the BPC of a sentence $s_{i}^{j}$ with its original speaker $c_{i}$ as its speaker prompt. For example, “Donald Trump: Make America great again” is denoted by ${BPC}_{c_{i}}(s_{i}^{j})$. The BPC of the same sentence $s_{i}^{j}$ with an alternative speaker prompt $c_{k}$, e.g. “Hillary Clinton: Make America great again” is denoted by ${BPC}_{c_{k}}(s_{i}^{j})$. Now, we define a “uniqueness” score for $s_{i}^{j}$ sentence as follows:


$$\text{S}\text{ENT}\text{U}\text{NIQ}(s_{i}^{j})=\left(\frac{1}{|C|-1}\sum_{c_{k}\in C\setminus \{c_{i}\}}{BPC}_{c_{k}}(s_{i}^{j})\right)-{BPC}_{c_{i}}(s_{i}^{j})$$

Intuitively, this means that the uniqueness of a sentence is defined as the difference between its BPC with its original speaker prompt and the average of its BPC scores with each of the |C|−1 alternative candidates as a speaker prompt. The greater this difference is, the higher the $\text{S}\text{ENT}\text{U}\text{NIQ}(s_{i}^{j})$ score is, suggesting that sentence $s_{i}^{j}$ is most likely to be said by the original speaker $c_{i}$ and not by the other candidates. Since debates include an opponent speaker in the transcript (e.g. Trump vs Biden), we exclude the opponent from the replacement candidates for sentences from that particular debate (i.e. C∖{Trump, Biden}), to avoid simulating unrealistic debates with only one speaker.

Then, the overall “uniqueness” score for a candidate $c_{i}$ is the average of the SENTUNIQ scores for each of $c_{i}$’s sentences in $S_{i}$:


$$\text{U}\text{NIQ}(c_{i})=\frac{1}{\left|S_{i}\right|}\sum_{s_{i}^{j}\in S_{i}}\text{S}\text{ENT}\text{U}\text{NIQ}(s_{i}^{j})$$

Again, the intuition here is that the larger this score, the greater the average difference is for sentence uniqueness, i.e. overall, speaker $c_{i}$’s sentences are not likely to be said by another candidate.

### Divisiveness lexicon

First, a vector space word model is used to populate a list of candidate divisive words. Then, we review and refine the candidate list through researcher annotations. The word vector model used is Gensim’s glove-wiki-gigaword-300 (28, 29).

Ten seed “divisive” terms are chosen manually by NLP and political science experts as common politically divisive English terms, before seeing the actual speech data. The ten seed terms used are: “stupid,” “dishonest,” “unamerican,” “idiot,” “deplorable,” “pathetic,” “immoral,” “disgrace,” “incompetent,” “foolish.” With these seed terms, 350 additional terms with the highest cosine similarity in the vector space model are added to the lexicon. Each of these initial 360 terms is analyzed by four researchers as “divisive” or “not divisive” based on the following criteria:

- No modifiers, e.g. “utterly,” “extremely”
- Should not include words that can often be used both divisively and nondivisively
- Should only include words that would be considered divisive in most political contexts

Only words that receive a majority of votes by the annotators are included in the final lexicon. The final size of the lexicon is 178 words. The entire lexicon can be found in the Supplementary material.

####  

##### Annotator agreement

Following (30), in order to analyze how often the annotators agree with each other, we additionally calculate the percentage of times the majority class has size 4 (all annotators agree), size 3 (all but one agree), and size 2 (even split). Table 5 shows these agreement values: 58.9% of the 360 initial terms had full annotator agreement, and 86.7% of these 360 have at least 3/4 annotators agreeing on the label. When limiting to the 178 terms that at least 3 out of 4 annotators agree are divisive, 69.1% have full agreement on the label (and 100% have majority agreement, by definition). So, the final terms include primarily instances that have high agreement scores.

**Table 5.** Percentage of majority class annotator agreement on binary labels (divisive? “yes” or “no”).

	Majority class size (%)		
Terms	four	three	two
360 initial	58.9	27.8	13.3
178 final	69.1	30.9	0.0

58.9% of the 360 initial terms had full annotator agreement, and 86.7% of these 360 have at least 3/4 annotators agreeing on the label. When limiting to the 178 terms that at least 3/4 annotators agree are divisive, 69.1% have full agreement on the label.

Since there are four annotators, we calculate Fleiss’ *κ* for interannotator agreement, as opposed to Cohen’s *κ* which is designed for two raters. For the initial 360 terms, we obtain a Fleiss’ *κ* of 0.54, which indicates a moderate level of agreement. Discussion of this score and why it may underestimate true agreement is included in the Supplementary material.

### Fightin’ words overlap metric

Monroe et al. (21) introduce a methodology for lexical feature selection that calculates odds ratios between of word probabilities between two related corpora. We use this method with the informative Dirichlet prior to obtain the FWs in a data type for each candidate.

**Fig. 9.**
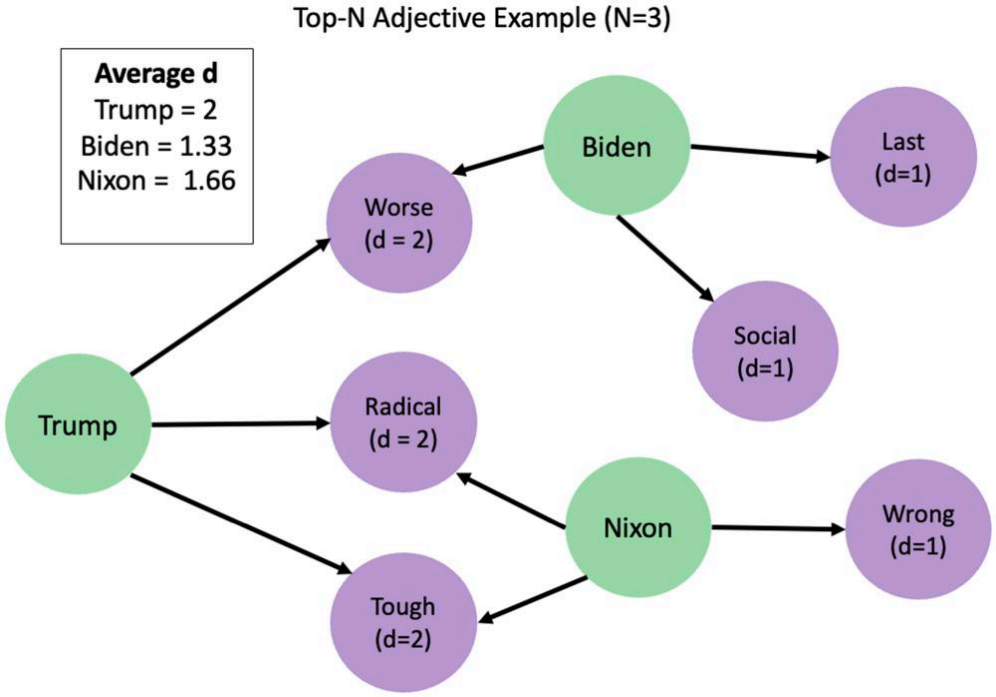
Conceptual example of how the FW overlap metric is calculated for N=3.

For each candidate s∈C, we specifically get the set of FWs between the words associated with their opponent mentions (${\mathrm{FW}}_{Y}(s)$) versus the set of those that are not (${\mathrm{FW}}_{N}(s)$). We then examine the top-*n* of these sets, respectively, ${\mathrm{FW}}_{Y}^{n}(s)$ and ${\mathrm{FW}}_{N}^{n}(s)$. For *Y* opponent mentions, we create a graph representation as follows (see Fig. 9):

- speaker nodes *S*, where each node corresponds to a candidate
- word nodes *W*, where *W* corresponds to the union of each candidate’s top-*n* (${\mathrm{FW}}_{Y}^{n}(s)$), i.e. $W=\underset{s\in C}{⋃}{\mathrm{FW}}_{Y}^{n}(s)$
- edges E(S,W), where edge *e* is added between s∈S and w∈W if *w* is in $FW_{Y}^{n}(s)$

The top-*n* FW overlap metric ($OM_{Y}^{n}(s)$) is then calculated as the


$$OM_{Y}^{n}(s)=\frac{1}{n}\sum_{\forall \;w\in FW_{Y}^{n}(s)}\text{deg}(w),$$

where deg(w) corresponds to the degree of the word node (# edges entering *w*). Likewise, for the set of words not associated with opponent mentions, we have


$$OM_{N}^{n}(s)=\frac{1}{n}\sum_{\forall \;w\in FW_{N}^{n}(s)}\text{deg}(w).$$

Equivalently, the overlap metric for *s* can be thought of as the average number of speaker sets $FW_{Y}^{n}(c_{i}),\;\forall \;c_{i}\in C$ that the top-*n* words of *s* appear in. A low $OM_{Y}^{n}(s)$ indicates that speaker *s* uses more distinct language to refer to opponents, while a higher score corresponds to opponent-referring language that is similar to that of other candidates.

## Supplementary Material

pgae431_Supplementary_Data

## Data Availability

The data underlying this article are available in the American Presidency Project at http://www.presidency.ucsb.edu/ws, and can be accessed publicly. For reproducibility, we release our data and code at https://github.com/ChicagoHAI/quantifying-unique-and-divisive-speech.

## Notes

a This finding is consistent with previous research showing that Trump uses language that is explicitly intended to evoke an emotional response from his revision audience (9), and that Trump’s speech patterns reveal his autocratic ambitions (10), antidemocratic views (11), a willingness to condone political violence (12), a commitment to populism (3), and low levels of analytic thinking (6). None of these scholars, however, have developed a lexicon of divisiveness, as we do in this paper.

b Additional data are used for model training. See Materials and Methods for details.

c Within these data, Donald Trump spoke in five debates (3,610 sentences), four SOTU addresses (1,471 sentences), and 28 campaign speeches (7,488 sentences) between the years of 2016–2020.

d There are considerably more sentences of nonopponent mentions, but only looking at top-*n* FW (across different thresholds) helps control for that difference.

e Some of these words, like “radical” and “liberal,” appear in the “polarization” dictionary of (17). Taken as a whole though, our divisive word lexicon contains very little overlap with that dictionary.

f
https://www.presidency.ucsb.edu/documents/presidential-documents-archive-guidebook/annual-messages-congress-the-state-the-union

g Versions of each model were also trained on unmasked data. The results from these models are consistent with our main findings and are presented in Supplementary material.

## Preprints

A preprint of this article is published at https://arxiv.org/abs/2401.01405.

## References

[1] Tulis J . 1987. The rhetorical presidency. Princeton University Press.

[2] Radford A, Narasimhan K, Salimans T, Sutskever I. Improving language understanding by generative pre-training. 2018.

[3] Cinar I, Stokes S, Uribe A. 2020. Presidential rhetoric and populism. Pres Stud Q. 50(2):240–263.

[4] Anderson K . 2018. How to talk Trump. *The Atlantic*.

[5] Frischling B . 2019. Not “stable genius” again, or please stop making us run this analysis.

[6] Jordan KN, Sterling J, Pennebaker JW, Boyd RL. 2019. Examining long-term trends in politics and culture through language of political leaders and cultural institutions. Proc Natl Acad Sci USA. 116(9):3476–3481. 30808741 10.1073/pnas.1811987116 PMC6397582

[7] Kayam O . 2018. The readability and simplicity of Donald Trump’s language. Political Stud Rev. 16(1):73–88.

[8] Zhong W . 2016. The candidates in their own words: A textual analysis of 2016 president primary debates.

[9] Hart R . 2020. Trump and us: what he says and why people listen. Cambridge: Cambridge University Press.

[10] Körner R, Overbeck JR, Körner E, Schütz A. 2022. How the linguistic styles of Donald Trump and Joe Biden reflect different forms of power. J Lang Soc Psychol. 41(6):631–658.

[11] Jamieson KH, Taussig D. 2017. Disruption, demonization, deliverance, and norm destruction: the rhetorical signature of Donald J. Trump. Polit Sci Q. 132(4):619–650.

[12] Ntontis E, Jurstakova K, Neville F, Haslam SA, Reicher S. 2024. A warrant for violence? an analysis of Donald Trump’s speech before the us capitol attack. Br J Soc Psychol. 63(1):3–19. 10.1111/bjso.12679. 37602507

[13] Woolley JT, Peters G [dataset] . 1999. The American presidency project. Santa Barbara, CA. Available from World Wide Web: http://www.presidency.ucsb.edu/ws.

[14] Chen SF, Beeferman D, Rosenfeld R. 1998. Evaluation metrics for language models.

[15] Melis G, Dyer C, Blunsom P. 2018. On the state of the art of evaluation in neural language models.

[16] Liang P, et al 2023. Holistic evaluation of language models. Trans Mac Learn Res. 10.48550/arXiv.2211.09110. 37230490

[17] Simchon A, Brady WJ, Van Bavel JJ. 2022. Troll and divide: the language of online polarization. PNAS Nexus. 1(1):pgac019. 36712799 10.1093/pnasnexus/pgac019 PMC9802075

[18] Sap M, et al 2022. Annotators with attitudes: how annotator beliefs and identities bias toxic language detection. In: Proceedings of the 2022 Conference of the North American Chapter of the Association for Computational Linguistics: Human Language Technologies. Seattle, United States: Association for Computational Linguistics. p. 5884–5906.

[19] Wulczyn E, Thain N, Dixon L. 2017. Ex machina: Personal attacks seen at scale. In: Proceedings of the 26th international conference on World Wide Web. p. 1391–1399. 10.1145/3038912.3052591.

[20] Brown P, Levinson SC. 1987. Politeness: some universals in language usage. vol. 4. Cambridge University Press.

[21] Monroe B, Colaresi M, Quinn K. 2009. Fightin’ words: lexical feature selection and evaluation for identifying the content of political conflict. Political Anal. 16.

[22] Radford A , *et al*. 2019. Language models are unsupervised multitask learners.

[23] Team G, et al 2024. Gemma: Open models based on gemini research and technology.

[24] Li Y , *et al*. 2023. Textbooks are all you need ii: phi-1.5 technical report, arXiv, arXiv:2309.05463, preprint: not peer reviewed. 10.48550/arXiv.2309.05463

[25] Lewandowsky S, Jetter M, Ecker UKH. 2020. Using the president’s tweets to understand political diversion in the age of social media. Nat Commun. 11(1):5764. 33173060 10.1038/s41467-020-19644-6 PMC7655817

[26] Kurtzleben D . 2023. Why Trump’s authoritarian language about ‘vermin’ matters.

[27] Falcon W, The PyTorch Lightning team. 2019. PyTorch lightning.

[28] Pennington J, Socher R, Manning C. 2014. GloVe: global vectors for word representation. In: Moschitti A, Pang B, Daelemans W, editors. Proceedings of the 2014 Conference on Empirical Methods in Natural Language Processing (EMNLP). Doha, Qatar: Association for Computational Linguistics p. 1532–1543.

[29] Řehůřek R, Sojka P. 2010. Software framework for topic modelling with large corpora. In: Proceedings of the LREC 2010 Workshop on New Challenges for NLP Frameworks. Valletta, Malta: ELRA. p. 45–50.

[30] Mohammad S, Turney P. 2010. Emotions evoked by common words and phrases: using mechanical Turk to create an emotion lexicon. In: Inkpen D, Strapparava C, editors. Proceedings of the NAACL HLT 2010 Workshop on Computational Approaches to Analysis and Generation of Emotion in Text. Los Angeles (CA): Association for Computational Linguistics. p. 26–34.

