# Supplementary material for: Quantifying the uniqueness and divisiveness of presidential discourse
Source: PNAS Nexus. 2024 Oct 7;3(10):pgae431. doi: 10.1093/pnasnexus/pgae431 (PMC11495212; doi:10.1093/pnasnexus/pgae431)
Supplement: pgae431_Supplementary_Data [file pgae431_supplementary_data.pdf]

Supporting Information for

Quantifying the Uniqueness and Divisiveness of Presidential Discourse

Karen Zhou, Alexander A. Meitus, Milo Chase, Grace Wang, Anne Mykland, William Howell, and Chenhao Tan

Chenhao Tan.
E-mail: chenhao@uchicago.edu

This PDF file includes:

Supporting text
Figs. S1 to S14
Tables S1 to S4
SI References

Supporting Information Text

LLM-based Uniqueness Robustness Checks.

BPC Recall that we employ bits-per-character (BPC), also known as bits-per-byte, as a measure of "predictability" of a sentence. Lower BPC values correspond to higher predictability. We use BPC instead of perplexity or loss directly to account for variation in tokenization techniques. BPC is the sum of cross-entropy losses of each token in a sentence, divided by the number of characters (bytes) in the sentences. Fig. S1 shows average sentence BPC per candidate (Fig. S1a) and aggregated by party over sentence length (Fig. S1b). While in campaigns, Trump is the most unpredictable among those candidates, he is ranks closer to the middle for predictability in debates and SOTU.

Correlation with Readability Metrics We calculate the Spearman correlation coefficients for our uniqueness metric with existing readability scores. Our metric has little to no correlation with readability, while different readability indexes show strong correlation with each other (Fig. S2). Fig. S2 also confirms that our metric is dintinct from sentence length.

Uniqueness scores over time Fig. S3 shows the uniqueness scores over time; in particular, the scores increased in recent years. We also present the uniqueness scores by year/term for debates (Fig. S4), SOTU (Fig. S5), and campaigns (Fig. S6). Overall, Trump is still consistently the most unique every year and term, compared to those of the other presidential candidates. For debates, he is slightly more unique in his first election cycle in 2016, whereas in campaigns, his second election cycle involves more distinctive speech than his first. For SOTU addresses, Trump's speech becomes more distinct in his second year onwards.

Top %-ile uniqueness Fig. S7 shows that, when comparing the uniqueness scores of the top decile of unique sentences for each speaker, we again find that Trump is the most unique speaker in all our samples of political speech.

LLM-based uniqueness results with unmasked model In the main paper, we present results using language models that are trained on data with named entities masked out with a `<ENT>` token, as identified by the spaCy NER tagger. This decision is made to prevent the model from learning patterns like "Only Trump mentions Biden's name during debates".

We also fine-tune each dataset's model on the data without masking named entities, with results presented in Fig. S8. These results are overall consistent with those of the masked model. Fig. S8a shows that Trump remains the most distinctive speaker in all data types, while Fig. S8b confirms still that the distinctiveness holds across all sentence lengths. Trump is still more similar in uniqueness to Democrats than his fellow Republicans (Fig. S8c).

Validation with other LLMs We validate our uniqueness metric implementation with other LLMs. Specifically, we recalculate scores with the Gemma-2B and Phi-1.5 (1.3B) base models (1, 2). These models are considerably larger than the 124M parameters of GPT-2 (3). Like our original setup up for GPT-2, we further train each model on all available corresponding data with a learning rate of 5e-5. Each model is trained with LoRA tuning for 15 epochs, using LLaMA-Factory (4). As shown in Fig. S9, while the exact scores differ, the trends are consistent with the main results: that is, Trump's speech is the most unique among the selection of Democratic and Republican presidential candidates.

Additional Divisive Word Lexicon Results.

Divisive Word Lexicon Table. S1 contains the 178 words in our proposed divisive word lexicon.

Usage over time In debates and campaigns, divisive language usage increased after 2012 (Fig. S10), which corresponds to the onset of Trump's candidacy. Indeed, we find that Trump uses the most divisive language of all candidates.

Usage by candidate Heatmaps of divisive word usage can provide a more granular look at the specific divisive language used by each candidate (Fig. S11). For example, in the debates dataset, it is notable that the frequency of use of the word "racist" has become used more by recent candidates like Trump and Biden. In the debates dataset some of Donald Trump's most frequently used words in this lexicon are those like "disgrace", "stupid", "filthy", "hate", and "racist" (Fig. S11a). In Donald Trump's SOTU addresses, the most frequently used divisive words are those like "corrupt", "vile", "foolish", "cruel", and "savage" (Fig. S11b). In campaign speeches, Donald Trump's most frequently used divisive words are those like "corrupt", "crazy", "stupid", and "dishonest" (Fig. S11c).

Utterances Using Divisive Words Excerpts of speech using divisive words can be found in Table. S2, Table. S3, and Table. S4 for debates, SOTU, and campaign speeches respectively.

Annotator Agreement For the initial 360 terms, we obtain a Fleiss' κ of 0.54, which indicates a moderate level of agreement. Since divisiveness is a somewhat subjective phenomenon, this moderate agreement is similar to that obtained on other subjective lexicons, such as the word-emotion association (average Fleiss' κ of 0.29) (5) and the initial terms in the polarization dictionary (Cohen's κ of 0.61) (6). Our final set of 178 terms has full agreement from the majority of annotators, but we still discuss the implications of our Fleiss' κ below.

Agreement is affected by several characteristics of divisiveness. Firstly , divisiveness is a spectrum and terms can be "a little" to "extremely" divisive. Annotators only give binary labels, so disagreement may be higher due to lack of nuance. Relatedly, the words are presented out of context; they may be more or less divisive depending on usage. However, identifying all possible contexts of our lexicon usage is costly and nontrivial. Finally, like the word-emotion association lexicon (5), our initial data is biased, with more "not divisive" terms than "divisive" terms (based on only 31.6% of the 360 terms receiving unanimous

agreement). Both Cohen's and Fleiss' κ are considered conservative in such settings with label imbalance (5, 7, 8). As such, these κ values may underestimate the true rater agreement for these cases.

Ultimately, we introduce the first such lexicon of divisiveness for text analysis. Usage of our lexicon may be augmented with additional strategies like dependency parsing and leveraging surrounding context.

Additional Opponent Mention Results.

Rate of opponent mentions The overall rates of sentences that mention opponents for debates, SOTU, and campaign speeches are 20.60%, 0.83%, and 6.95% respectively. Fig. S12 shows the distribution of sentences containing opponent mentions among the candidate speakers. Trump has the highest rate of opponent mentions in debates.

Fightin' Words overlap We present additional plots for the Fightin' Words overlap metric, for different top-n adjectives in debates (see Fig. S13). In particular, for top-5, 10, and 25 adjective Fightin' Words, Trump has the lowest overlap in adjectives he uses in association with opponent mentions. These trends are consistent with those presented in the main paper.

Relating uniqueness, divisiveness, and opponent mentions.. Fig. S14a shows that in debates and campaign speeches, sentences that use divisive language tend to be more unique as well. The Spearman correlation coefficient between divisiveness and uniqueness is 0.01 for debates and campaigns (p <0.05 in campaigns and p =0.30 in debates). For sentences containing opponent mentions in debates, Fig. S14b shows that such utterances tend to be more distinctive and Fig. S14c shows that they tend to have higher divisive word usage as well. The Spearman correlation for opponent mentions is 0.05 and 0.02 (p <0.05) between uniqueness and divisiveness, respectively.

References

1. G Team, et al., Gemma: Open models based on gemini research and technology. *arXiv preprint arXiv:2403.08295* (2024).
2. Y Li, et al., Textbooks are all you need ii: **phi-1.5** technical report. *arXiv preprint arXiv:2309.05463* (2023).
3. A Radford, et al., Language models are unsupervised multitask learners (2019).
4. Y Zheng, et al., Llamafactory: Unified efficient fine-tuning of 100+ language models. *arXiv preprint arXiv:2403.13372* (2024).
5. SM Mohammad, PD Turney, Crowdsourcing a word-emotion association lexicon (2013).
6. A Simchon, WJ Brady, JJ Van Bavel, Troll and divide: the language of online polarization. *PNAS Nexus* **1**, pgac019 (2022).
7. RL Brennan, DJ Prediger, Coefficient kappa: Some uses, misuses, and alternatives. *Educ. Psychol. Meas.* **41**, 687–699 (1981).
8. WD Perreault, LE Leigh, Reliability of nominal data based on qualitative judgments. *J. Mark. Res.* **26**, 135–148 (1989).

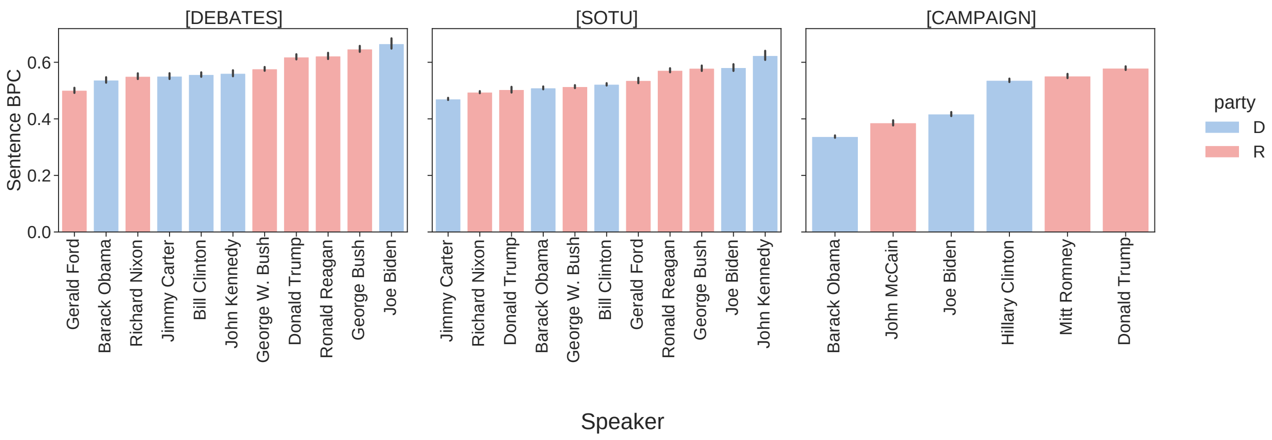

(a) Average sentence BPC per candidate (error bars represent 95%-confidence intervals)

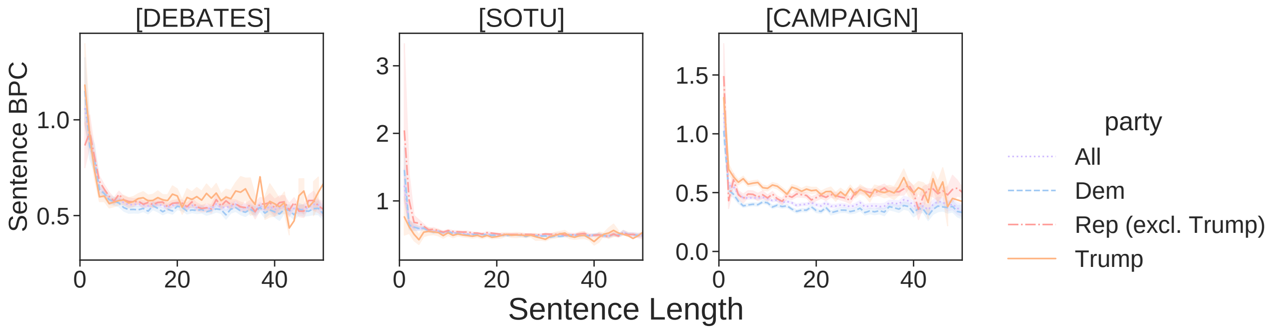

(b) Sentence BPC across sentence length

Fig. S1. Average sentence BPC across each data type. In campaigns, Trump is the most unpredictable among those candidates; however, he is ranks closer to the middle for predictability in debates and SOTU

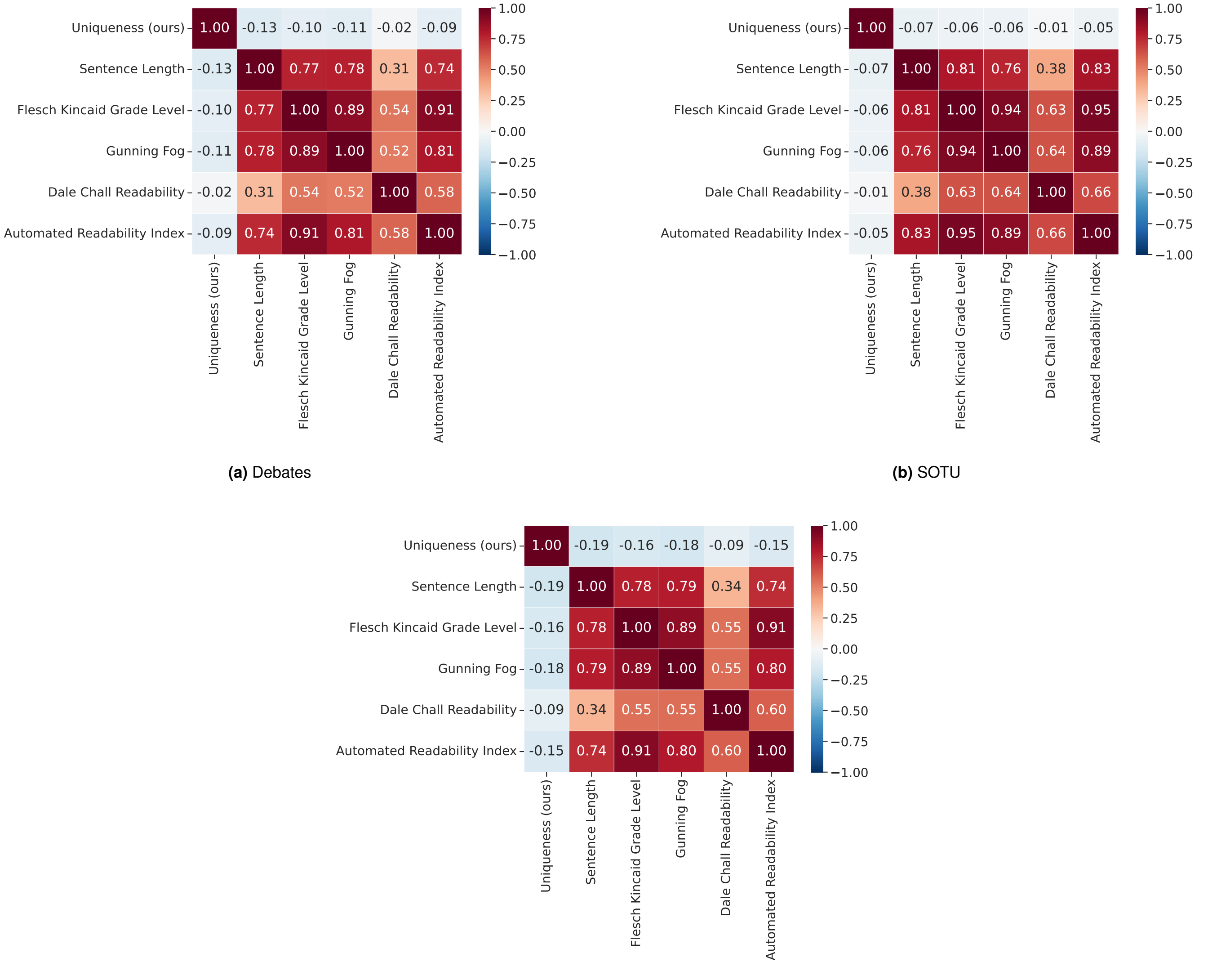

(a) Debates

(b) SOTU

(c) Campaign

Fig. S2. Heatmaps of Spearman Correlation Coefficients between our uniqueness metric and existing readability scores (FKGL, Gunning Fog, Dale Chall, and ARI). There is little to no correlation between our score and readability; in contrast, there is strong correlation between the different readability indexes.

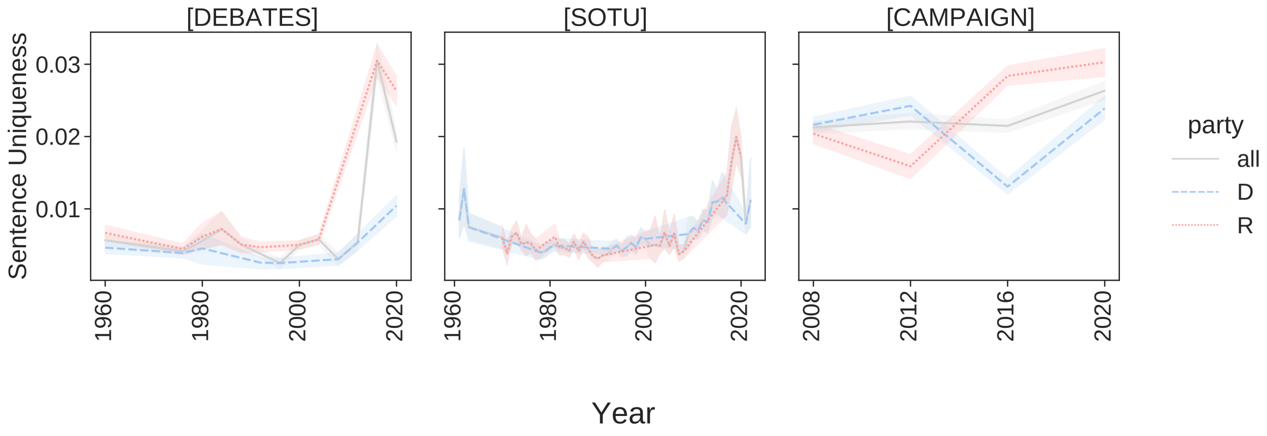

Fig. S3. Uniqueness score over time, aggregated by political party.

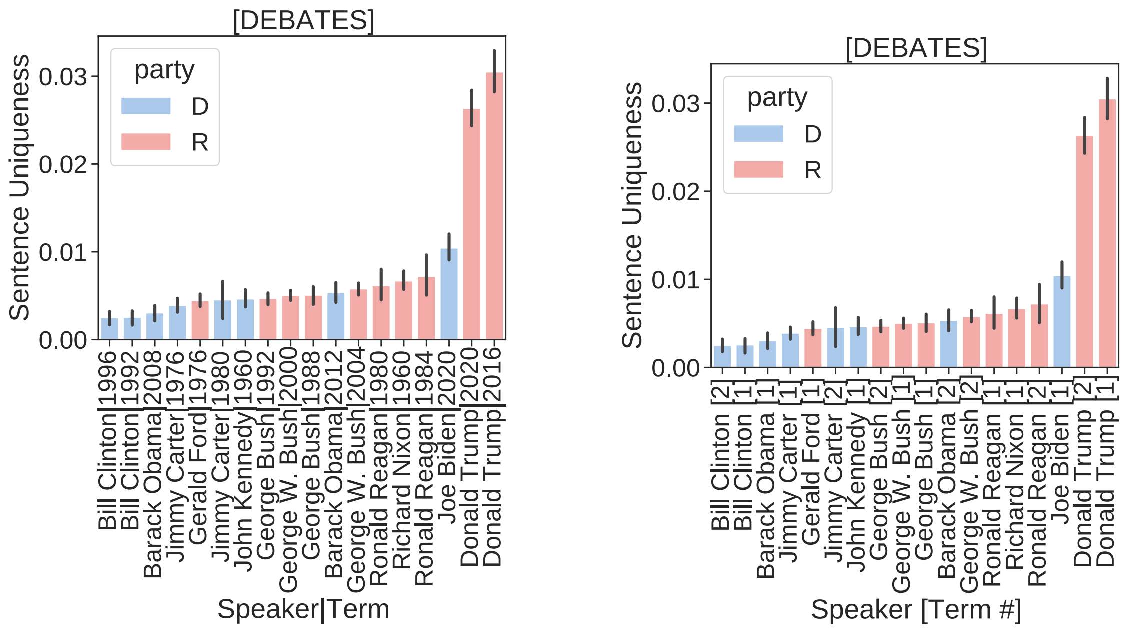

(a) Debates by election year

(b) Debates by term #

Fig. S4. For debates, Trump is consistently most unique for each term that he runs for president (2016, 2020). He is slightly more unique in his first election cycle, in 2016. The error bars represent 95%-confidence intervals.

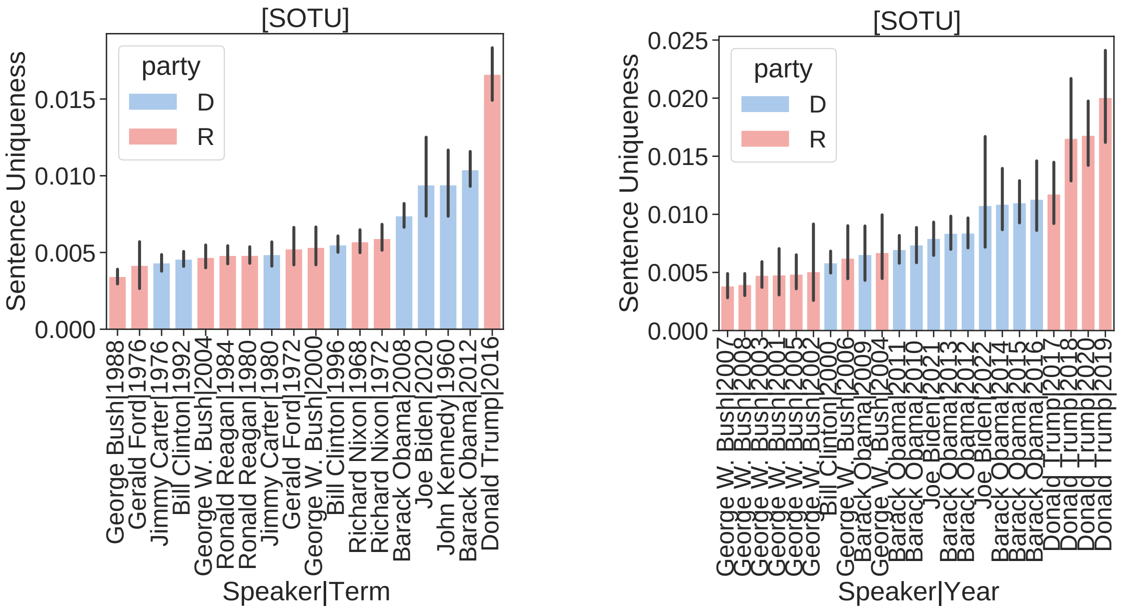

(a) SOTU by term

(b) SOTU by year

Fig. S5. For SOTU, Trump is consistently most unique throughout all years of his presidency. His speech distinctiveness increases after the first year of his term. The error bars represent 95%-confidence intervals.

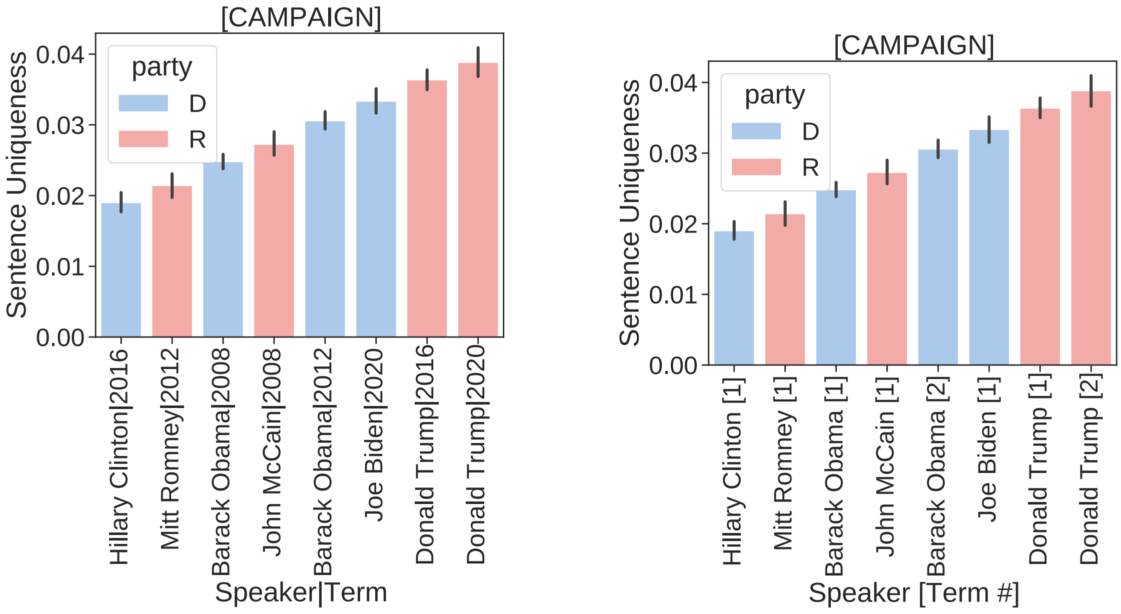

(a) Campaign by election year

(b) Campaign by term #

Fig. S6. For campaign speeches, Trump is consistently most unique for each term that he runs for president (2016, 2020). His campaign speech in his second election cycle is slightly more unique than his first time. The error bars represent 95%-confidence intervals.

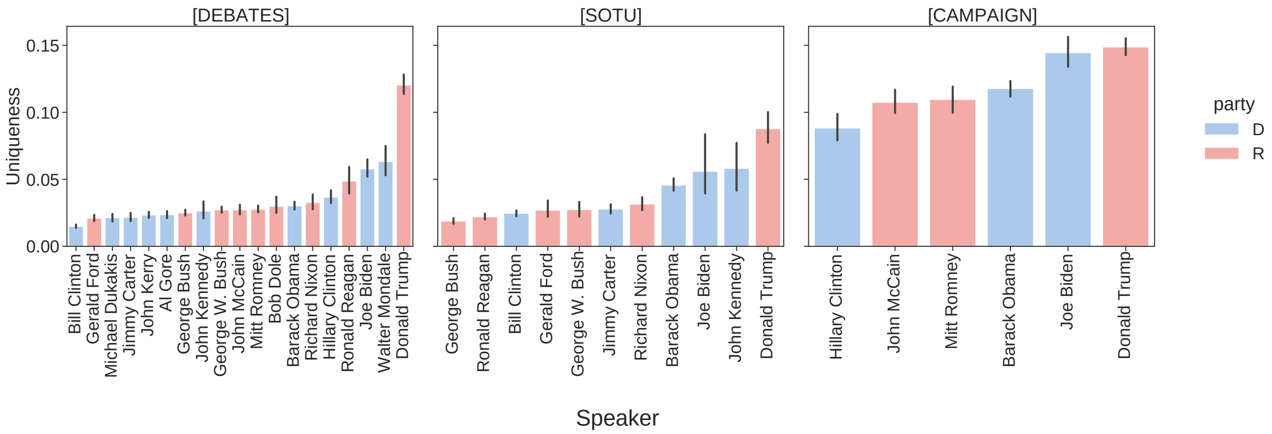

Fig. S7. Uniqueness score by candidate, of sentences within the top 10-ile of scores for each candidate (the error bars represent 95%-confidence intervals). Note that Trump continues to be most unique in all datasets.

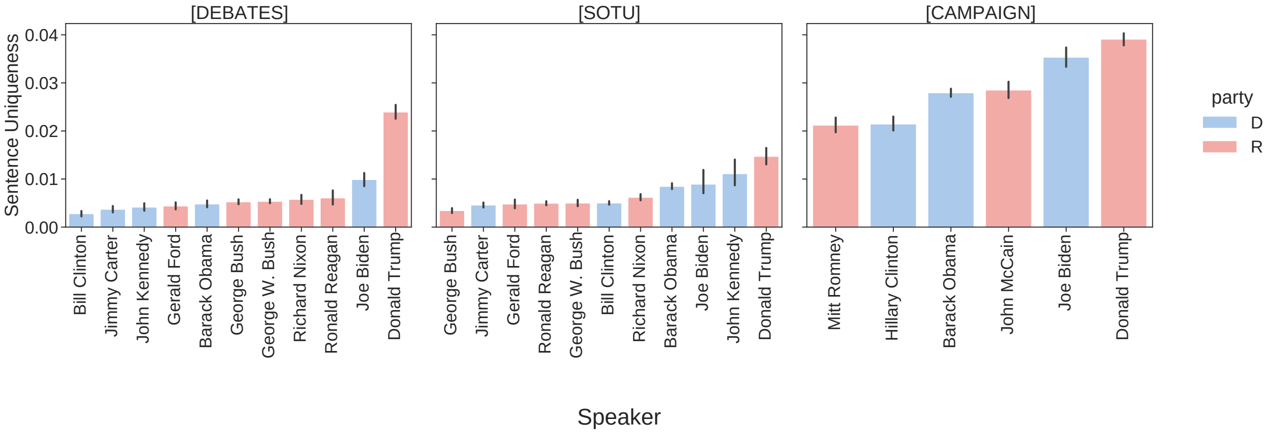

(a) Overall score by candidate

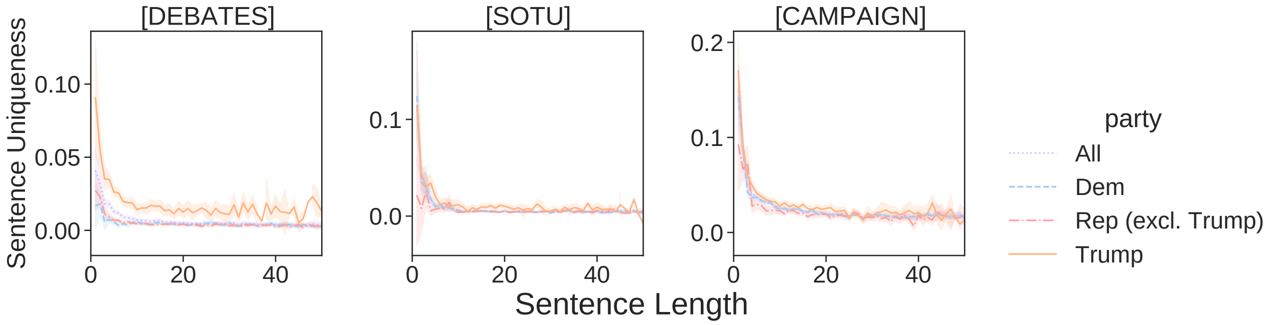

(b) Scores by sentence length

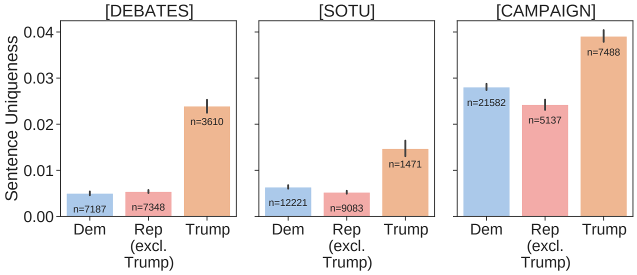

(c) Uniqueness by party

Fig. S8. Average sentence uniqueness for each speaker, across all data types, using the UNMASKED model (the error bars represent 95%-confidence intervals). Trump is still the most unique speaker in all types of speeches.

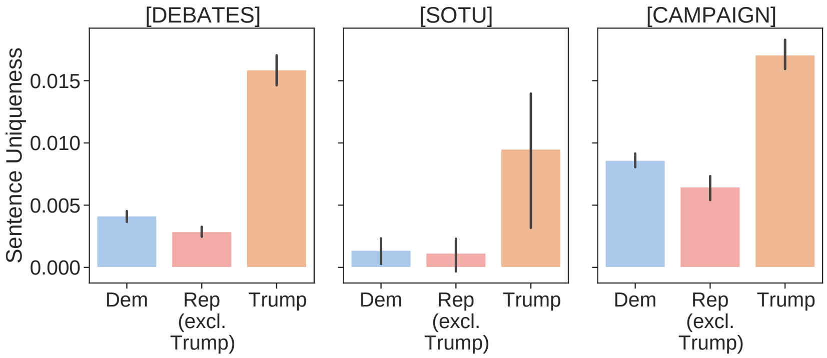

(a) Gemma 2B

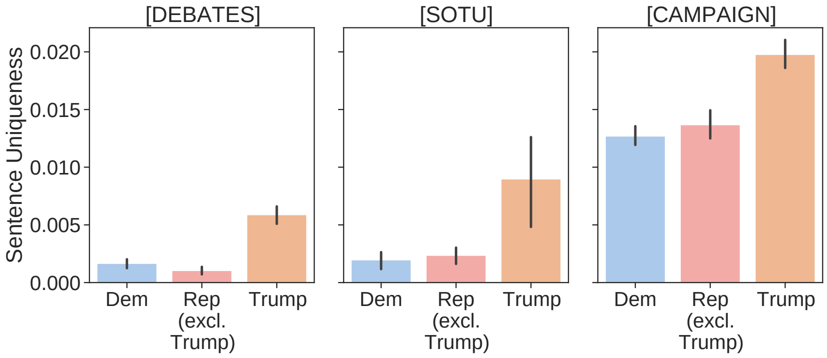

(b) Phi-1.5

Fig. S9. Validation of the LLM-based uniqueness metric with alternative LLMs. The resulting trends are consistent with original findings that Trump's speech is more unique than other Democratic and Republican presidential candidates.

stupid*,	disloyal,	liar,	fools,
dishonest*,	ashamed,	vindictive,	heinous,
unamerican*,	illogical,	careless,	thuggish,
idiot*,	distasteful,	wretched,	impulsive,
deplorable*,	naive,	hateful,	detestable,
pathetic*,	heartless,	bigoted,	demented,
immoral*,	crass,	bastards,	traitor,
disgrace*,	repugnant,	undemocratic,	hypocrites,
incompetent*,	sinful,	feckless,	filthy,
foolish*,	unpatriotic,	cowardice,	conceited,
irresponsible,	abhorrent,	pompous,	traitors,
shameful,	obnoxious,	mindless,	loathsome,
disgraceful,	childish,	spineless,	barbarous,
disgusting,	thoughtless,	arrogance,	irrational,
hypocritical,	stupidity,	rude,	savages,
idiotic,	demeaning,	treasonous,	gullible,
unprofessional,	untrustworthy,	vulgar,	obscene,
ridiculous,	awful,	disgusted,	repulsive,
despicable,	egotistical,	insufferable,	delusional,
unethical,	deluded,	sleazy,	insult,
outrageous,	dumb,	fool,	scoundrel,
arrogant,	greedy,	perverse,	abominable,
inexcusable,	boorish,	scurrilous,	deplore,
ignorant,	barbaric,	insolent,	mockery,
shameless,	tasteless,	grotesque,	perversion,
disrespectful,	foolishness,	devious,	bumbling,
absurd,	cruel,	incompetence,	dopey,
ludicrous,	amoral,	buffoon,	inane,
reprehensible,	pretentious,	unintelligent,	nauseating,
unconscionable,	hypocrite,	undisciplined,	brainless,
scandalous,	revolting,	odious,	incorrigible,
contemptible,	reckless,	insane,	exploitive,
inept,	manipulative,	liars,	gutless,
unworthy,	nonsensical,	depraved,	dishonesty,
appalling,	idiots,	crazy,	unqualified,
laughable,	sexist,	moronic,	conniving,
disingenuous,	intolerable,	uncouth,	promiscuous,
cowardly,	uncivilized,	petulant,	degrading,
callous,	egregious,	ugly,	racist,
unjust,	duplicitous,	elitist,	ruthless,
indefensible,	undignified,	frivolous,	perverted,
foolhardy,	atrocious,	hypocrisy,	diabolical,
selfish,	corrupt,	dastardly,	betrayal
preposterous,	contemptuous,	incapable,	
deceitful	vile	tyrannical	

Table S1. The 178 words in our divisive word lexicon, which are verified as being divisive by the majority of 4 annotators. Our original ten seed words are denoted with a *.

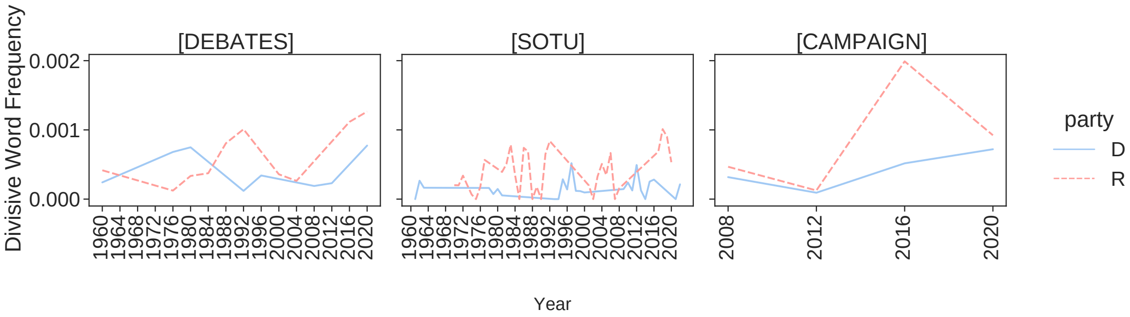

Fig. S10. Frequency of usage over time

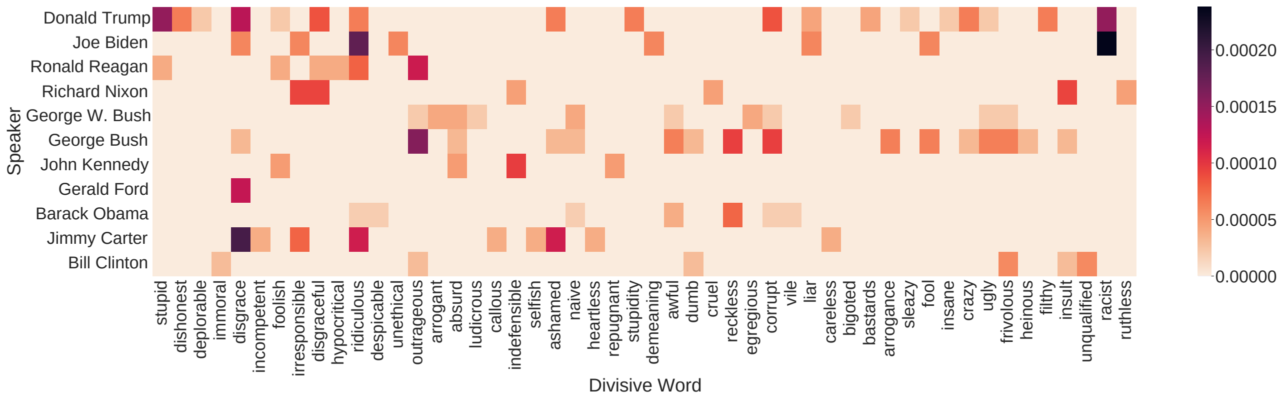

(a) Debates

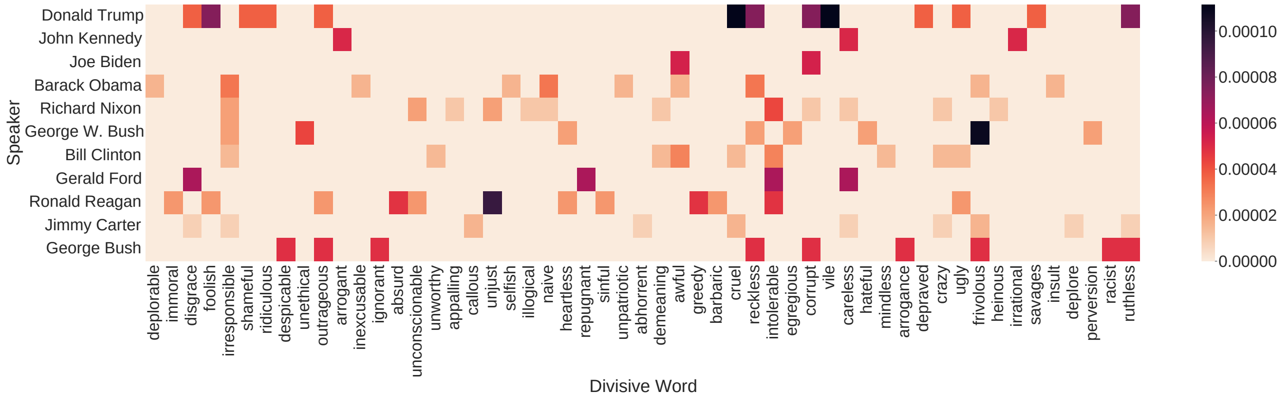

(b) SOTU

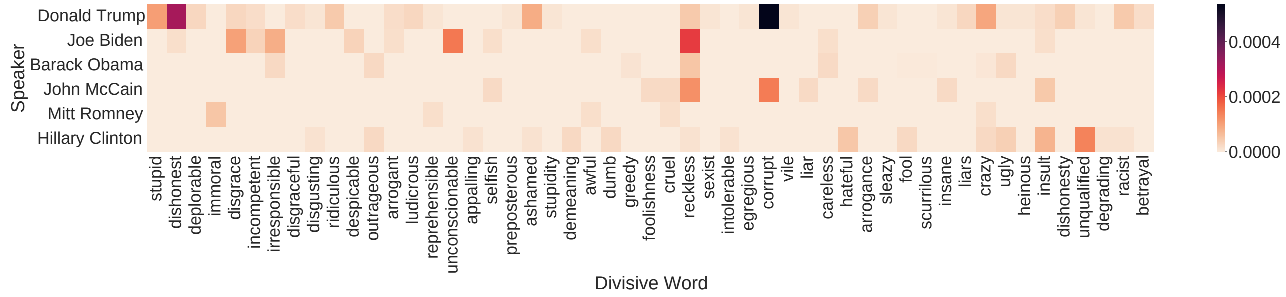

(c) Campaign

Fig. S11. Heatmaps showing divisive word utterance frequency, normalized over all words spoken, by speaker.

Year	Example Sentence(s) w/ Context
2020	**Joe Biden:** I'm talking about the Biden plan. . .<br>**Donald Trump:** Where they want to rip down buildings. . .<br>**Other:** [to Biden] Let him go for a minute, and then you can go.<br>**Donald Trump:** And rebuild the building.<br>**Joe Biden:** No.<br>**Donald Trump:** It's the **dumbest**-<br>**Joe Biden:** That is not, that is not. . .<br>**Donald Trump:** ... most **ridiculous**. . .
2020	**Joe Biden:** Russia is paying you a lot.<br>**Joe Biden:** China is paying a lot.<br>**Joe Biden:** And your hotels and all your businesses all around the country, all around the world.<br>**Joe Biden:** And China's building a new road to a new gas a golf course you have overseas.<br>**Joe Biden:** So what's going on here?<br>**Joe Biden:** Why don't you release your tax return or stop talking about **corruption**?
2020	**Donald Trump:** We have to go back to the core values of this country.<br>**Donald Trump:** They were teaching people that our country is a horrible place.<br>**Donald Trump:** It's a **racist** place.<br>**Donald Trump:** And they were teaching people to hate our country.<br>**Donald Trump:** And I'm not going to allow that to happen.<br>**Joe Biden:** Nobody's doing that.<br>**Other:** Vice President Biden.<br>**Joe Biden:** Nobody's doing that.<br>**Joe Biden:** He's the **racist**.
2020	**Donald Trump:** I mean, they can say anything.<br>**Donald Trump:** It's a very– it makes me sad because I am the least **racist** person.<br>**Donald Trump:** I can't even see the audience because it's so dark, but I don't care who's in the audience.<br>**Donald Trump:** I'm the least **racist** person in this room.<br>**Other:** OK.<br>**Other:** Vice President Biden, let me ask you, very quickly, and then I have a follow up question for you.<br>**Joe Biden:** Abraham Lincoln.<br>**Joe Biden:** Here is one of the most **racist** presidents we've had in modern history.<br>**Joe Biden:** He pours fuel on every single **racist** fire, every single one.
2016	**Donald Trump:** Well, all of these bad leaders from ISIS are leaving Mosul.<br>**Donald Trump:** Why can't they do it quietly?<br>**Donald Trump:** Why can't they do the attack, make it a sneak attack, and after the attack is made, inform the American public that we've knocked out the leaders, we've had a tremendous success?<br>**Donald Trump:** People leave.<br>**Donald Trump:** Why do they have to say we're going to be attacking Mosul within the next four to six weeks, which is what they're saying?<br>**Donald Trump:** How **stupid** is our country?
2016	**Donald Trump:** But the leaders that we wanted to get are all gone because they're smart.<br>**Donald Trump:** They say, what do we need this for?<br>**Donald Trump:** So Mosul is going to be a wonderful thing.<br>**Donald Trump:** And Iran should write us a letter of thank you, just like the really **stupid** the **stupidest** deal of all time, a deal that's going to give Iran absolutely nuclear weapons.
2016	**Hillary Clinton:** And we should demand that Donald release all of his tax returns so that people can see what are the entanglements and the financial relationships that he has...<br>**Other:** We're going to get to that later.<br>**Other:** Secretary Clinton, you're out of time.<br>**Hillary Clinton:** ... with the Russians and other foreign powers.<br>**Other:** Mr. Trump?<br>**Donald Trump:** Well, I think I should respond, because so **ridiculous**.
1976	**Gerald Ford:** On the other hand, when you have a bill of that magnitude, with those many provisions, a President has to sit and decide if there is more good than bad.<br>**Gerald Ford:** And from the analysis that I have made so far, it seems to me that that tax bill does justify my signature and my approval.<br>**Other:** Governor Carter, your response.<br>**Jimmy Carter:** Well, Mr. Ford is changing considerably his previous philosophy.<br>**Jimmy Carter:** The present tax structure is a **disgrace** to this country.
1988	**George Bush:** In terms of negative campaigning, you know, I don't want to sound like a kid in the schoolyard: he started it.<br>**George Bush:** But take a look at the Democratic convention take a look at it.<br>**George Bush:** Do you remember the Senator from Boston chanting out there and the ridicule factor from that lady from Texas that was on there; I mean, come on, this was just **outrageous**.

Table S2. [DEBATES] Example usages of words from our divisive lexicon throughout debates.

Year	Example Sentence(s) w/ Context
2022	**Joe Biden:** Putin is now isolated from the world more than he has ever been.<br>**Joe Biden:** Together, along with our allies, we are right now enforcing powerful economic sanctions.<br>**Joe Biden:** We're cutting off Russia's largest banks from the international financial system; preventing Russia's Central Bank from defending the Russian ruble, making Putin's $630 billion war fund worthless.<br>**Joe Biden:** We're choking Russia's access to technology that will sap its economic strength and weaken its military for years to come.<br>**Joe Biden:** Tonight I say to the Russian oligarchs and the **corrupt** leaders who have bilked billions of dollars off this violent regime: No more.
2020	**Donald Trump:** The terrorist responsible for killing Sergeant Hake was Qasem Soleimani, who provided the deadly roadside bomb that took Chris's life.<br>**Donald Trump:** Soleimani was the Iranian regime's most **ruthless** butcher, a monster who murdered or wounded thousands of American servicemembers in Iraq.
2019	**Donald Trump:** On Friday, it was announced that we added another 304,000 jobs last month alone, almost double the number expected.<br>**Donald Trump:** An economic miracle is taking place in the United States, and the only thing that can stop it are **foolish** wars, politics, or ridiculous, partisan investigations.
2018	**Donald Trump:** I am asking Congress to address the fundamental flaws in the terrible Iran nuclear deal.<br>**Donald Trump:** My administration has also imposed tough sanctions on the communist and socialist dictatorships in Cuba and Venezuela.<br>**Donald Trump:** But no regime has oppressed its own citizens more totally or brutally than the **cruel** dictatorship in North Korea.
2018	**Donald Trump:** In April, this will be the last time you will ever file under the old and very broken system, and millions of Americans will have more take-home pay starting next month—a lot more.<br>**Donald Trump:** We eliminated an especially **cruel** tax that fell mostly on Americans making less than $50,000 a year, forcing them to pay tremendous penalties simply because they couldn't afford Government-ordered health plans.
2017	**Donald Trump:** We cannot allow a beachhead of terrorism to form inside America.<br>**Donald Trump:** We cannot allow our Nation to become a sanctuary for extremists.<br>**Donald Trump:** That is why my administration has been working on improved vetting procedures, and we will shortly take new steps to keep our Nation safe and to keep those out who will do us harm.<br>**Donald Trump:** As promised, I directed the Department of Defense to develop a plan to demolish and destroy ISIS, a network of lawless savages that have slaughtered Muslims and Christians, and men and women and children of all faiths and all beliefs.<br>**Donald Trump:** We will work with our allies, including our friends and allies in the Muslim world, to extinguish this **vile** enemy from our planet.
2016	**Barack Obama:** But after years now of record corporate profits, working families won't get more opportunity or bigger paychecks just by letting big banks or big oil or hedge funds make their own rules at everybody else's expense.<br>**Barack Obama:** Middle class families are not going to feel more secure because we allowed attacks on collective bargaining to go unanswered.<br>**Barack Obama:** Food stamp recipients did not cause the financial crisis; **recklessness** on Wall Street did.
2012	**Barack Obama:** In 2008, the house of cards collapsed.<br>**Barack Obama:** We learned that mortgages had been sold to people who couldn't afford or understand them.<br>**Barack Obama:** Banks had made huge bets and bonuses with other people's money.<br>**Barack Obama:** Regulators had looked the other way or didn't have the authority to stop the bad behavior.<br>**Barack Obama:** It was wrong, it was **irresponsible**, and it plunged our economy into a crisis that put millions out of work, saddled us with more debt, and left innocent, hard-working Americans holding the bag.
1991	**George Bush:** Last year, our friends and allies provided the bulk of the economic costs of Desert Shield.<br>**George Bush:** And now, having received commitments of over $40 billion for the first 3 months of 1991, I am confident they will do no less as we move through Desert Storm.<br>**George Bush:** But the world has to wonder what the dictator of Iraq is thinking.<br>**George Bush:** If he thinks that by targeting innocent civilians in Israel and Saudi Arabia, that he will gain advantage, he is dead wrong.<br>**George Bush:** If he thinks that he will advance his cause through tragic and **despicable** environmental terrorism, he is dead wrong.
1982	**Ronald Reagan:** Contrary to some of the wild charges you may have heard, this administration has not and will not turn its back on America's elderly or America's poor.<br>**Ronald Reagan:** Under the new budget, funding for social insurance programs will be more than double the amount spent only 6 years ago.<br>**Ronald Reagan:** But it would be **foolish** to pretend that these or any programs cannot be made more efficient and economical.

Table S3. [SOTU] Example usages of words from our divisive lexicon throughout SOTU addresses. In general, there are fewer uses of divisive words during SOTU speeches.

Year	Example Sentence(s) w/ Context
2020	**Joe Biden:** The last thing you need is a President who ignores you, looks down at you, who just doesn't understand you.<br>**Joe Biden:** Like President Trump.<br>**Joe Biden:** His **reckless** personal conduct since his diagnosis, the destabilizing effect it's having on our government, is **unconscionable**.
2020	**Joe Biden:** I'm so grateful to have earned the UA's endorsement — and to have 355,000 proud plumbers, pipefitters, and more behind me.<br>**Joe Biden:** I also want to thank Rick for sharing his story with us today—and for being part of our convention this year where he shared his story with America.<br>**Joe Biden:** Farmers all across this country have been gutted by President Trump's broken promises and **reckless** trade war.
2020	**Donald Trump:** This is the most important election in the history of our country.<br>**Donald Trump:** Six months ago I was saying, "Well, how do you compare with the last one?"<br>**Donald Trump:** I don't know.<br>**Donald Trump:** That was important.<br>**Donald Trump:** The fact is, this is the single most important election in the history of our country.<br>**Donald Trump:** And sleepy Joe Biden's made a **corrupt** bargain.
2016	**Donald Trump:** Now, Bernie Sanders should be angry right?<br>**Donald Trump:** Shouldn't he be angry?<br>**Donald Trump:** Now, I'll tell you what.<br>**Donald Trump:** The system is rigged.<br>**Donald Trump:** The system is rigged.<br>**Donald Trump:** I've been saying it for a—it's rigged, and we're gonna straighten it out.<br>**Donald Trump:** But the system is rigged.<br>**Donald Trump:** Hillary is not the victim; the American people are the victims of this system.<br>**Donald Trump:** So **corrupt** in so many ways.
2016	**Donald Trump:** I want the entire **corrupt** Washington establishment to hear and to heed the words we all will be saying right now.<br>**Donald Trump:** When we win on November 8th, we are going to Washington, D.C. and we are going to drain the swamp.<br>**Donald Trump:** Gonna drain the swamp.<br>**Donald Trump:** We're gonna drain the swamp, folks.<br>**Donald Trump:** We're gonna drain that swamp.<br>**Donald Trump:** Another important issue for Americans is integrity in journalism.<br>**Donald Trump:** These people are among the most **dishonest** people I've ever met, spoken to, done business with.<br>**Donald Trump:** These are the most **dishonest** people.
2016	**Donald Trump:** Our trade deals, we lose $800 billion a year on trade.<br>**Donald Trump:** We have trade deficits.<br>**Donald Trump:** Think of that.<br>**Donald Trump:** Who negotiates these deals?<br>**Donald Trump:** You know who does?<br>**Donald Trump: Stupid** people.<br>**Donald Trump: Stupid** people.<br>**Donald Trump:** With very **stupid** leadership.
2016	**Donald Trump:** We will terminate NAFTA and get a much better deal for our workers if we can't renegotiate it properly.<br>**Donald Trump:** We're gonna get a better deal for our workers and for our companies.<br>**Donald Trump:** Because we cannot continue to be the people led by **stupid** people.
2016	**Hillary Clinton:** We should honor the men and women in uniform who fight for our country.<br>**Hillary Clinton:** That's why I was so appalled when Donald Trump tweeted that the new effort underway to push the terrorists out of the key city of Mosul is already, and I quote him, "a total disaster" and that our country is, again a quote, "looking **dumb**."<br>**Hillary Clinton:** Really?<br>**Hillary Clinton:** He's declaring defeat before the battle has even started.<br>**Hillary Clinton:** He's proving once again he is **unqualified** to be commander in chief of our military.
2016	**Hillary Clinton:** But this is not new.<br>**Hillary Clinton:** I know I'm reaching out to Republicans and Independents as well as Democrats because I want to be the president for all Americans.<br>**Hillary Clinton:** And, when you think about it, what he said at the convention, 'I alone can fix it,' runs counter to who we are as Americans.<br>**Hillary Clinton:** We work together.<br>**Hillary Clinton:** So there are many reasons why I think it is fair to conclude that Donald Trump is **unqualified** and unfit to be president.
2008	**John McCain:** In a time of trouble and danger for our country, who will put our country first?<br>**John McCain:** In 21 months, during hundreds of speeches, town halls and debates, I have kept my promise to level with you about my plans to reform Washington and get this country moving again.<br>**John McCain:** As a senator, I've seen the **corrupt** ways of Washington in wasteful spending and other abuses of power.
2008	**John McCain:** In his three short years in the Senate, he has requested nearly a billion dollars in pork projects for his state - a million dollars for every day he's been in office.<br>**John McCain:** Far from fighting earmarks in Congress, Senator Obama has been an eager participant in this **corrupt** system.

Table S4. [CAMPAIGN] Example usages of words from our divisive lexicon throughout campaigns.

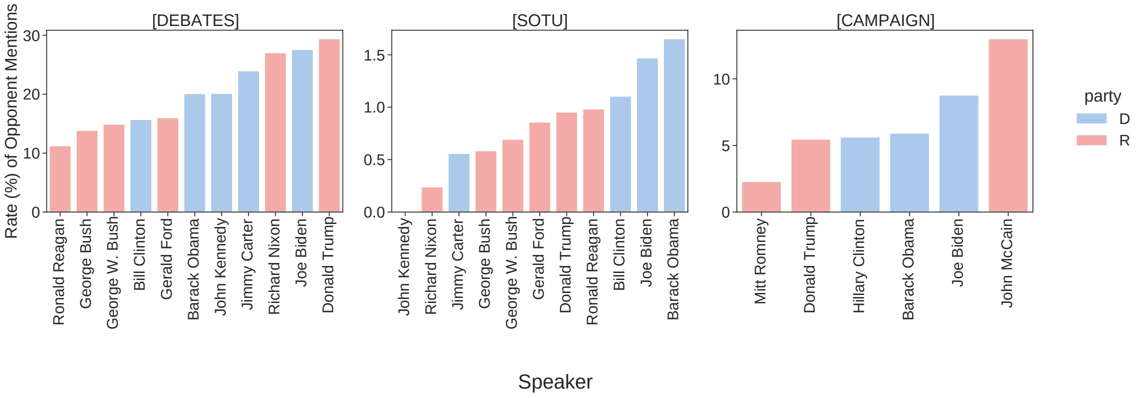

Fig. S12. Distribution of sentences containing opponent mentions among all speakers, across the data types. Note the different rates for debates vs. SOTU. Trump has the highest rate of opponent mentions in debates.

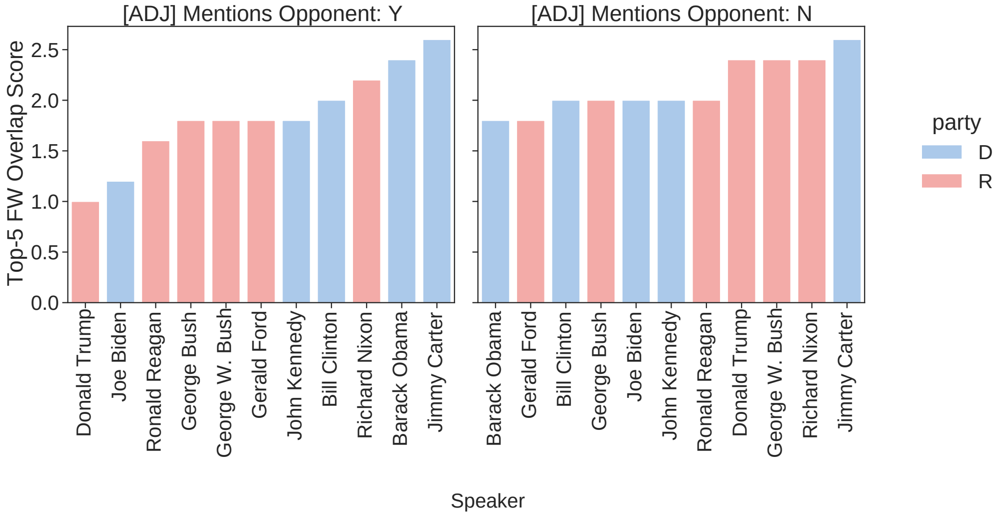

(a) Top-5 FW overlap score in debates for each candidate

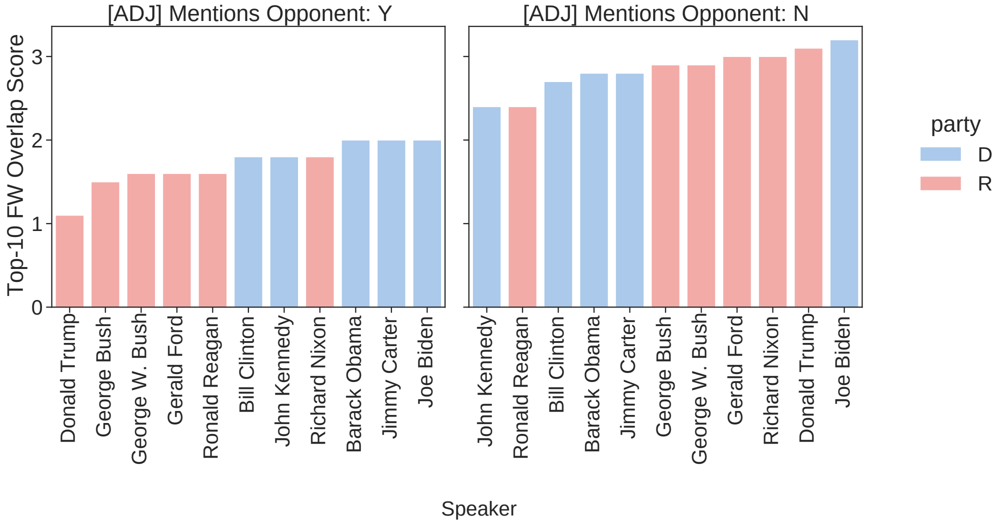

(b) Top-10 FW overlap score in debates for each candidate

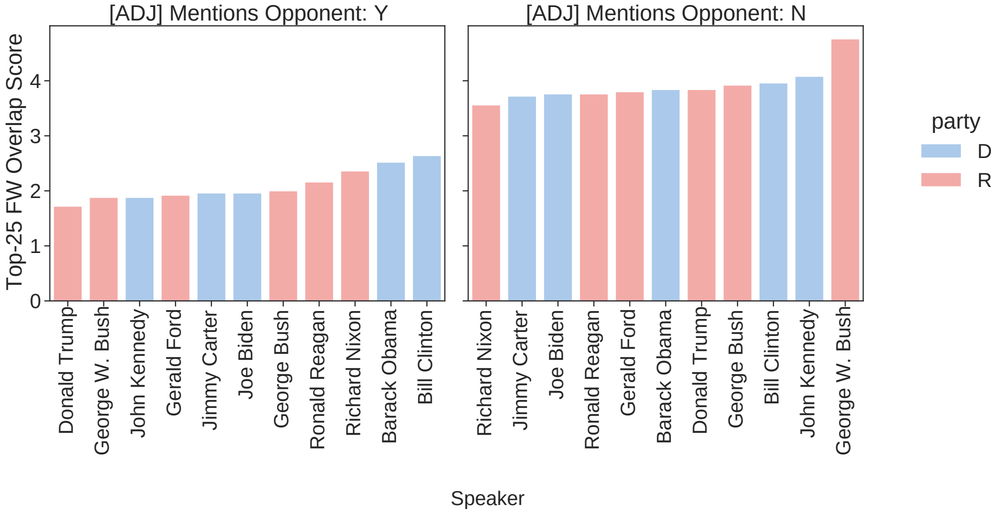

(c) Top-25 FW overlap score in debates for each candidate

Fig. S13. Fightin' Words overlap results from debates. Trump's FW associated with opponent mentions generally have the lowest overlap in adjective usage compared to other candidates, which is another indication that his language is distinctive.

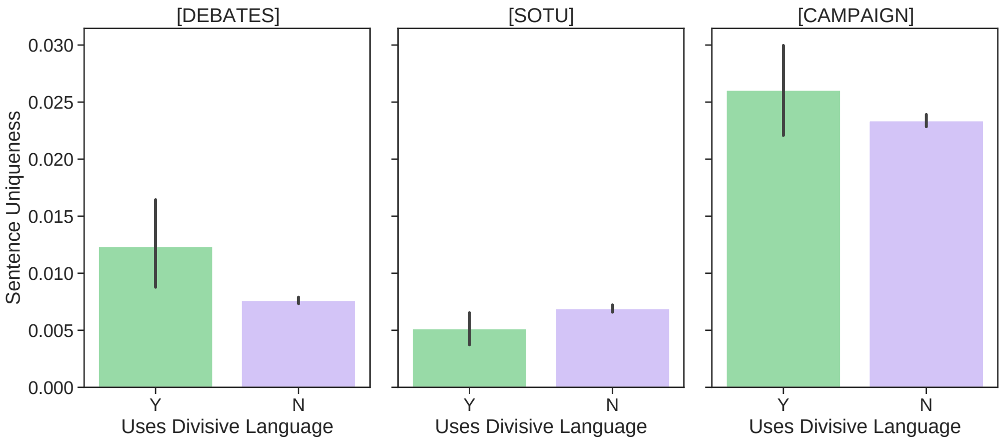

(a) Sentence uniqueness across divisive language usage, for each type of speech.

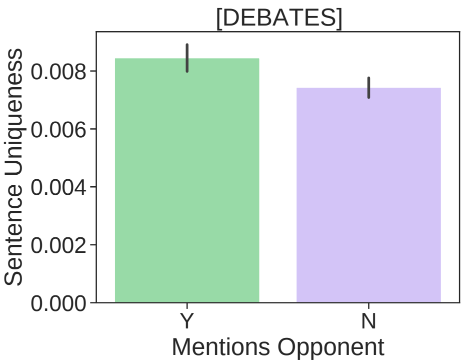

(b) Sentence uniqueness across opponent mentions, in debates.

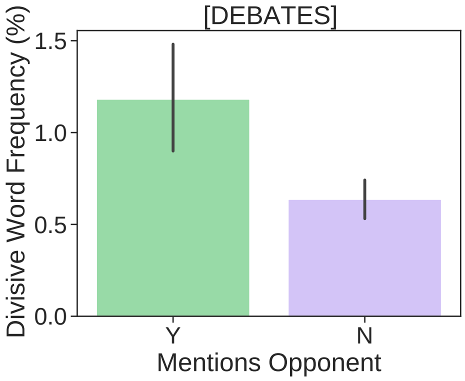

(c) Divisive language frequency across opponent mentions, in debates

Fig. S14. Pairwise analysis of our three proposed metrics, aggregated globally across all speakers. Fig. S14a shows that in debates and campaign speeches, sentences that use divisive language tend to be more unique as well. For sentences containing opponent mentions in debates, Fig. S14b shows that such utterances tend to be more distinctive and Fig. S14c shows that they tend to have higher divisive word usage as well.
